# The Cost-Effectiveness of Biologics for the Treatment of Rheumatoid Arthritis: A Systematic Review

**DOI:** 10.1371/journal.pone.0119683

**Published:** 2015-03-17

**Authors:** Jaana T. Joensuu, Saara Huoponen, Kalle J. Aaltonen, Yrjö T. Konttinen, Dan Nordström, Marja Blom

**Affiliations:** 1 Faculty of Pharmacy, University of Helsinki, Helsinki, Finland; 2 Institute of Clinical Medicine, University of Helsinki, Helsinki, Finland; 3 Helsinki University Central Hospital, Helsinki, Finland; Glasgow University, UNITED KINGDOM

## Abstract

**Background and Objectives:**

Economic evaluations provide information to aid the optimal utilization of limited healthcare resources. Costs of biologics for Rheumatoid arthritis (RA) are remarkably high, which makes these agents an important target for economic evaluations. This systematic review aims to identify existing studies examining the cost-effectiveness of biologics for RA, assess their quality and report their results systematically.

**Methods:**

A literature search covering Medline, Scopus, Cochrane library, ACP Journal club and Web of Science was performed in March 2013. The cost-utility analyses (CUAs) of one or more available biological drugs for the treatment of RA in adults were included. Two independent investigators systematically collected information and assessed the quality of the studies. To enable the comparison of the results, all costs were converted to 2013 euro.

**Results:**

Of the 4890 references found in the literature search, 41 CUAs were included in the current systematic review. While considering only direct costs, the incremental cost-effectiveness ratio (ICER) of the tumor necrosis factor inhibitors (TNFi) ranged from 39,000 to 1 273,000 €/quality adjusted life year (QALY) gained in comparison to conventional disease-modifying antirheumatic drugs (cDMARDs) in cDMARD naïve patients. Among patients with an insufficient response to cDMARDs, biologics were associated with ICERs ranging from 12,000 to 708,000 €/QALY. Rituximab was found to be the most cost-effective alternative compared to other biologics among the patients with an insufficient response to TNFi.

**Conclusions:**

When 35,000 €/QALY is considered as a threshold for the ICER, TNFis do not seem to be cost-effective among cDMARD naïve patients and patients with an insufficient response to cDMARDs. With thresholds of 50,000 to 100,000 €/QALY biologics might be cost-effective among patients with an inadequate response to cDMARDs. Standardization of multiattribute utility instruments and a validated standard conversion method for missing utility measures would enable better comparison between CUAs.

## Introduction

Rheumatoid arthritis (RA) is a chronic autoimmune disease with the prevalence of 0.2–1% among adult population in Europe and North-America [1]. RA affects physical health causing pain, stiffness, progressive joint destruction and physical disability. Medical treatment, joint replacement surgery and productivity losses due to sick leave and early retirements lead to significant expenses for society [2]. The treatment target of RA is remission or low disease activity and the medication initially comprises conventional disease-modifying antirheumatic drugs (cDMARDs) such as methotrexate (MTX), sulphasalazine (SSZ), hydroxychloroquine (HCQ) and leflunomide (LEF), low-dose prednisolone and their combinations [3]. However, not all patients achieve remission or low disease activity with cDMARDs due to intolerance or lack of effectiveness. Biologic disease-modifying antirheumatic drugs (bDMARDs), also known as biologics, cover TNF inhibitors (TNFi) (adalimumab (ADA) (Humira, AbbVie Ltd.), certolizumab pegol (CER) (Cimzia, UCB Pharma SA), etanercept (ETN) (Enbrel, Pfizer Ltd.), golimumab (GOL) (Simponi, Janssen Biologics B.V), infliximab (IFX) (Remicade, Janssen Biologics B.V.)) and agents based on other mechanisms of action (abatacept (ABT) (Orencia, Bristol-Myers Squibb Pharma EEIG), anakinra (ANA) (Kineret, Biovitrum AB), rituximab (RTX) (MabThera, Roche Registration Ltd) and tocilizumab (TOC) (RoActemra, Roche Registration Ltd.)). Biologics have proven to be an effective treatment for RA, but because of the high price, they are recommended only for patients with insufficient response or intolerance to cDMARDs [3–6].

Economic evaluations provide information on the benefits and costs of these expensive treatments to aid the optimal utilization of limited healthcare resources [7]. Cost-effectiveness analysis (CEA) is the most typical form of economic evaluation for health care interventions. In CEA, costs and effectiveness of two or more treatments are compared. The costs are measured in monetary units and effectiveness in natural units, for example in life years or pain free days. Cost-utility analysis (CUA) is a subtype of CEA, applying quality adjusted life years (QALY) as a measure of effectiveness. The primary outcome measure in CUAs is incremental cost-effectiveness ratio ICER, which describes the ratio of the additional costs of a treatment (compared to an alternative) to QALYs gained. An ICER is not reported if one treatment is both cheaper and more effective than another, e.g. if it is dominant.

Biologics for RA are an important target for economic evaluations because of the associated high costs. Previous systematic reviews suggest that biologics might be cost-effective at the willingness to pay (WTP) threshold of 50,000–100,000 $/QALY among patients with insufficient treatment response to cDMARD but not in cDMARD naïve patients [8–10]. However, these reviews involve some weaknesses such as lack of quality assessment [9], insufficient reporting of study characteristics [8] or omission of between-biologics comparison [10]. The aim of our systematic review is to identify all existing studies examining the cost-utility of one or more biologics for RA in adults, assess their quality and report their results systematically.

## Methods

### Literature search

We performed a literature search aiming to identify existing CUAs assessing the cost-effectiveness of biologics for treatment of RA. The search covering Medline, SCOPUS (including EMBase), Cochrane library (Database of Abstracts of Reviews of Effects, Health Technology Assessment Database, Cochrane Database of Systematic Reviews, NHS Economic Evaluation Database, Cochrane Central Register of Controlled Trials and Cochrane Methodology Register), ACP Journal club and Web of science was executed in March 2013 using a search strategy developed with a librarian. The search strategy included terms describing study design (CUA), intervention (Biologics) and patients (RA) in different spellings. The complete search strategy for PubMed is presented in S1 File.

No time or language restrictions were made to the literature search. The number of non-English publications was used to investigate the existence of a language bias and publication bias was assessed based on the number of conference abstract published as full-text.

### Study selection

All references identified by the literature search were imported to reference management software (Refworks), where duplicate records were removed. Of the remaining references, the CUAs of one or more currently available biologics for the treatment of RA in adults were selected using a pre-defined inclusion and exclusion criteria (S1 Table). The evaluation for inclusion was conducted independently by two persons (JJ and KA) at first by titles and afterwards by full-text. In case of disagreement, a third opinion (MB) was requested. Studies without active comparison treatment (cDMARDs or other biologics) or QALYs as measure of effectiveness were excluded from this systematic review. Reporting of ICER was required, if applicable. Studies published only as conference abstracts and articles without English full-text were excluded.

### Data collection

The Data on patients, interventions, controls, study design (country, perspective, time horizon, the year of resource utilization, included costs, discount rate, the source of effectiveness, the instrument for utility measures, study funding) and outcomes were extracted using a Microsoft Excel—based collection form. Two assessors (JJ and SH) independently extracted the data and discrepancies were resolved by consulting the third investigator (MB). Due to limited time and resources, authors were not contacted for complementary information.

### Quality assessment

As currently recommended, the quality of economic evaluations included was assessed using the British Medical Journal (BMJ) checklist and in addition, the Philips`checklist for modelling studies [11–13]. Two investigators (JJ and SH) assessed the quality of the studies independently and the third investigator (MB) was consulted when necessary. BMJ checklist involves 35 items and Philips’ checklist 57 items. Quality scores based on fulfilment of items and average percentages of the applicable criteria met were calculated. To assess the relative quality of the studies we divided studies in three categories (good, adequate and poor quality) ranking them by using the average percentages.

### Representation of results

The quantitative synthesis of the results of the studies included is not possible owing to heterogeneous study designs. Results of the CUAs included were stratified into five subgroups by type of drug used, previous treatments and response to them, and the comparator treatment as follows: 1) Biologics for cDMARDs naive patients, 2) Biologics compared with cDMARD in patients with an inadequate response to one or several cDMARDs, 3) Biologics compared with other biologics among patients with an inadequate response to cDMARDs, 4) Biologics compared with cDMARDs among patients with an inadequate response to TNFi(s) and 5) Biologics compared with other biologics among patients with an inadequate response to TNFi(s). Further, CUAs were stratified according to adequateness of the comparator treatment. Adequate comparator was defined as a cDMARD not used before [3].

To enable a comparison of the results, all of the reported costs were converted to euro using the European Central Bank exchange rates (http://sdw.ecb.europa.eu) and adjusted to the price level of the year 2013 using the price index of Health care expenditure in Finland (Statistics Finland). ICERs including only direct costs were considered primary results due to differences in the ways indirect costs (e.g. productivity losses) were calculated in studies. In addition, ICERs including both direct and indirect costs were presented as secondary outcomes, if reported in the original studies.

## Results

**Fig 1:**
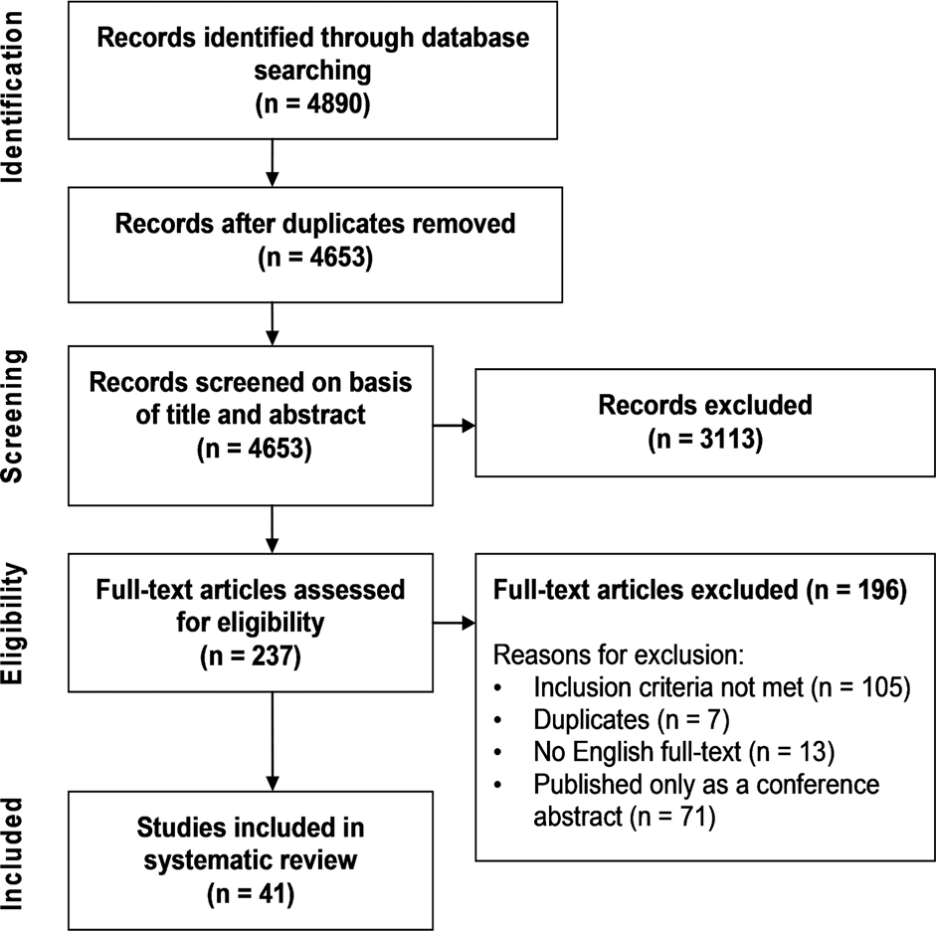
Flow chart of the study selection process.

Altogether, 4653 non-duplicate references were identified with the literature search, of which 3113 were excluded during title and abstract screening (Fig. 1). After the assessment of 237 full-text articles, 41 were included in the current review. A majority of the studies excluded by full-text assessment did not meet the inclusion criteria (105 studies) or were published only as conference abstracts (71 studies). The list of the articles excluded after full-text assessment is displayed in S2 File.

### Characteristics of studies included in the current review

The 41 CUAs included were published 2002–2013 [14–54]. One study was based on empiric cost and effectiveness data from a randomised controlled trial (RCT) [19], two on observational data [16,37] while the remaining 38 studies used a modelling approach with multiple data sources [14,15,17,18,20–36,38–54]. In 33 of the 38 modelling studies effectiveness estimates were derived from one or more RCTs, while five modelling studies applied effectiveness obtained from national registers. A summary of the characteristics of the CUAs included is shown in Table 1.

**Table 1:** Characteristics of the studies included in the current review.

Study, Year of publication, Country	Patients	Biologic treatment(s)	Comparator	Perspective	Time horizon	Study type	Source of effectiveness	Instrument for utility measures	Discount rate*
Bansback et al. 2005, Sweden [41]	Moderate to severe RA, inadequate response to 2 cDMARDs	ADA+MTX or ADA or ETN+MTX or ETN or IFX+MTX	cDMARD	Policy maker	Lifetime	Patient-level transition state model	RCTs	HUI-3 converted from HAQ, QoL = 0.76–0.28 x HAQ + 0.05 x FEMALE	3%
Barbieri et al. 2005, UK [51]	Severe RA, inadequate response to MTX	IFX+MTX	MTX	Payer (UK NHS)	Lifetime (model), 1 and 2 years and lifetime (treatment)	Markov model	RCT	VAS	Costs 6%, benefits 1.5%
Barton et al. 2004, UK [50]	RA, inadequate response to SSZ or MTX	ETN / IFX➔ cDMARDs	cDMARDs: GST ➔ AZA ➔ D-PEN ➔HCQ➔ LEF ➔ CSA ➔ MTX/CSA➔ Palliation	Payer (UK NHS)	Lifetime	Individual sampling model	RCTs	EQ-5D converted from HAQ, QoL = 0.862–0.327 x HAQ	Costs 6%, benefits 1.5%
Brennan et al. 2004, UK [49]	RA, inadequate response to at least 2 cDMARDs (MTX, SSZ)	ETN➔ cDMARDs	cDMARDs: GST➔LEF➔CSA	Payer	Lifetime	Individual patient-level simulation model	RCT	EQ-5D converted from HAQ, QoL = 0.86–0.20 x HAQ	Costs 6%, benefits 1.5%
Brennan et al. 2007, UK [15]	RA, inadequate response to at least 2 cDMARDs	TNFi (ETN, IFX, ADA)	cDMARDs	Payer (UK NHS)	Lifetime	Individual sampling model	British Registry (BSRBR)	EQ-5D converted from HAQ	Costs 6%, benefits 1.5%
Brodszky et al. 2010, Hungary [34]	Moderate to severe RA, inadequate response to cDMARDs and at least1 TNFi	RTX	1.) MTX, 2.) Another TNFi	Health care provider	Lifetime (model), 2 infusions and 3 years (treatment)	Markov model	RCTs	EQ-5D converted from HAQ	5%
CADTH 2010, Canada [48]	RA, inadequate response to at least 2 cDMARDs	ADA or ETN or IFX or GOL or ABT or Optimal sequence of biologics	MTX	Health care provider	5 years	Markov model	MTC	HUI-3 converted from HAQ, QoL = 0.76–0.28 x HAQ + 0.05 x FEMALE	Not stated
Chen et al. 2006, UK [28]	1.) Early RA, no previous cDMARDs and 2.) RA, inadequate response to at least 2 cDMARDs (SSZ, MTX)	IFX+MTX / ADA+MTX / ETN+MTX / ETN / ADA➔cDMARDs or cDMARDs➔ IFX+MTX / ETN+MTX / ADA+MTX / ETN /ADA	cDMARDs:(MTX)➔ MTX+SSZ ➔ MTX+SSZ+HCQ ➔ LEF ➔ GST ➔ AZA (CSA ➔ CSA+MTX ➔ D-PEN or cDMARDs: MTX+SSZ+HCQ ➔ LEF ➔ GST ➔ AZA ➔ CSA ➔ CSA+MTX ➔ D-PEN	Payer (UK NHS)	Lifetime	Individual sampling model	Meta-analysis in same report	EQ-5D converted from HAQ, QoL = 0.862–0.327 x HAQ	Costs 6%, benefits 1.5%
Chiou et al. 2004 [47]	Moderate to severe RA	ETN+MTX or ETN or ADA+MTX or ADA or ANA+MTX or ANA or IFX+MTX	Comparison of biologics	Payer	1 year	Decision analytic model	RCTs	VAS converted from ACR20, ACR50, ACR70 and no ACR responses	-
Clark et al. 2004, UK [22]	RA, inadequate response to cDMARDs and TNFi (SSZ, MTX, HCQ, (GST), LEF, ETN, IFX)	ANA➔cDMARDs or cDMARDs➔ANA	cDMARDs: (GST)➔ AZA➔CSA➔ MTX+CSA	Payer (UK NHS)	Lifetime	Individual sampling model	Meta-analysis in same report	EQ-5D converted from HAQ, QoL = 0.862–0.327 x HAQ	Costs 6%, benefits 1.5%
Coyle et al. 2006, Canada [46]	RA, no response to cDMARDs (MTX, MTX+SSZ, MTX+SSZ+HCQ)	IFX+MTX / ETN➔GST or GST➔IFX+MTX / ETN	GST	Third party payer (Ministry of Health)	5 years	Markov model	Systematic review in same report	EQ-5D converted from HAQ	5%
Davies et al. 2009, USA [21]	Early RA (< 3 years), no previous MTX	ADA+MTX / ETN / IFX+MTX ➔ cDMARDs or ADA+MTX➔ ETN➔ cDMARDs	cDMARDs: MTX ➔ MTX+HCQ ➔ LEF ➔ GST ➔ Palliation	Payer	Lifetime	Individual patient-level simulation model	Several RCTs	HUI-3 converted from HAQ, QoL = 0.76–0.28 x HAQ	3%
Diamantopoulos et al. 2012, Italy [33]	RA, inadequate response to cDMARDs	TOC+MTX ➔ biologics: (ADA+MTX ➔ RTX+MTX ➔ ABA+MTX ➔ Palliation)	ETN+MTX ➔ biologics	Payer	Lifetime	Individual patient-level simulation model	MTC	EQ-5D converted from HAQ, QoL = 0.82–0.11 x HAQ—0.07 x HAQ²	3%
Farahani et al. 2006, Canada [37]	RA	ETN + cDMARD	cDMARD (MTX, SSZ, HCQ etc.)	Societal	1 year	Observational analysis, no modelling used	RCT and observational study (efficacy vs. effectiveness data)	EQ-5D converted from HAQ, QoL = 0.862–0.327 x HAQ	-
Finckh et al. 2009, USA [27]	Early RA (< 3 months), no previous cDMARDs	1.) cDMARDs ➔ 1.TNFi+MTX ➔ 2.TNFi+MTX➔ 3.TNFi 2.)1.TNFi+MTX ➔ 2.TNFi+MTX➔ 3.TNFi ➔ cDMARDs 3.)NSAID ➔ cDMARDs ➔ 1.TNFi+MTX ➔ 2.TNFi+MTX ➔ 3.TNFi	Comparison of treatment 3 strategies containing TNFi	Health care provider, societal	Lifetime	Individual sampling model	Meta-analysis	EQ-5D converted from HAQ	3%
Hallinen et al. 2010, Finland [54]	Severe RA, no response to TNFi	RTX+MTX / ADA+MTX / ETN+MTX / IFX+MTX/ ABT+MTX➔ cDMARDs or Optimal sequence of biologics	cDMARDs: GST ➔ CSA+MTX	Societal	Lifetime (up to the age of 100 years)	Patient-level Markov model	RCTs	HUI-3 converted from HAQ, QoL = 0.76–0.28 x HAQ + 0.05 x FEMALE	3%
Jobanputra et al. 2002, UK [32]	RA, no response at least 2 cDMARDs (SSZ, MTX)	ETN / IFX+MTX ➔ cDMARDs or cDMARDs➔ ETN / IFX+MTX	cDMARDs: GST ➔ AZA ➔ D-PEN ➔ HCQ ➔ LEF ➔ CSA ➔ CSA+MTX	Payer (UK NHS)	Lifetime	Individual sampling model	Meta-analysis in same report	EQ-5D converted from HAQ	Costs 6%, benefits 1.5%
Kielhorn et al. 2008, UK [31]	RA, inadequate response to 2 cDMARDs and a TNFi	RTX ➔ MTX ➔ cDMARDs or RTX+MTX ➔ ADA+MTX ➔ IFX+MTX ➔ cDMARDs	cDMARDs: LEF ➔ GST ➔ CSA ➔ MTX or ADA+MTX ➔ IFX+MTX ➔ cDMARDs	Payer (UK NHS)	Lifetime	Patient-level Markov model	RCTs	HUI-3 converted from HAQ, QoL = 0.76–0.28 x HAQ + 0.05 x FEMALE	3,5%
Kobelt et al. 2003, UK & Sweden [52]	Advanced RA, no response to MTX	IFX+MTX	MTX	Not stated	10 years (model), 1 and 2 years (treatment)	Markov model	RCT	EQ-5D converted from HAQ	UK: Costs 6%, benefits 1.5%; Sweden: 3%
Kobelt et al. 2004, Sweden [16]	RA, inadequate response to at least 2 cDMARDs, including MTX	TNF (IFX, ETN)	Baseline (DMARD)	Societal	1 year	Observational analysis, no modelling used	Observational study	EQ-5D	-
Kobelt et al. 2005, Sweden [36]	RA, inadequate response to cDMARD (excluding MTX)	ETN+MTX or ETN	MTX	Societal	5 and 10 years (model); 2, 5 and 10 years (treatment)	Patient-level Markov model	RCT	EQ-5D converted from HAQ	3%
Kobelt et al. 2011, Sweden [38]	Early RA, no previous MTX	ETN+MTX ➔ Half-dose ETN+MTX ➔ cDMARD / 2. biologic	MTX ➔ cDMARD / biologic	Societal	10 years	Patient-level Markov model	RCT	EQ-5D converted from HAQ	3%
Lekander et al. 2010, Sweden [26]	RA, inadequate response to at least 2 cDMARDs	IFX + cDMARD	cDMARD	Societal	20 years	Markov cohort model	Registry (STURE)	EQ-5D converted from HAQ	3%
Lekander et al. 2013, Sweden [25]	1.) RA, inadequate response to at least 2 cDMARDs or 2.) RA, inadequate response to a TNFi	TNFi (ADA, IFX, ETN) + cDMARD or TNFi or ETN+cDMARD or ETN	cDMARD	Societal	20 years	Markov cohort model	Registry (Swedish Rheumatology Register)	EQ-5D converted from HAQ	3%
Lindgren et al. 2009, Sweden [45]	RA, inadequate response to a TNFi	RTX ➔ 2.TNFi (ADA, ETN, IFX)	2. TNFi ➔ 3. TNFi	Societal	Lifetime	Discrete event simulation model	RCT and Registry (SSTAG)	EQ-5D converted from HAQ and DAS 28	3%
Malottki et al. 2011, UK [53]	RA, inadequate response to a TNFi	ADA / ETN / IFX / RTX / ABT ➔ cDMARDs	cDMARDs: LEF ➔ GST ➔ CSA ➔ AZA	Payer (UK HNS)	Lifetime	Individual sampling model	Meta-analysis	EQ-5D converted from HAQ, QoL = 0.804–0.203 x HAQ—0.045 x HAQ²	3,5%
Marra et al. 2007, Canada [44]	RA, refractory to standard therapy	IFX+MTX	MTX	Societal	10 years	Patient-level Markov model	RCT	HUI-2, HUI-3, EQ-5D and SF6D converted from HAQ	3%
Merkesdal et al. 2010, Germany [18]	RA, inadequate response to ETN	RTX+MTX ➔ ADA+MTX ➔ IFX+MTX ➔ GST ➔ CSA ➔ MTX	ADA+MTX ➔ IFX+MTX ➔ GST ➔ CSA ➔ MTX	Payer	Lifetime	Patient-level Markov model	RCTs	HUI-3 converted from HAQ, QoL = 0.76–0.28 x HAQ + 0.05 x FEMALE	3,5%
Nguyen et al. 2012, USA [24]	Moderate to severe RA, moderate or no response to MTX	ADA+MTX / IFX+MTX / CER+MTX / GOL+MTX ➔ TOC	ETN+MTX ➔ TOC / MTX ➔ TOC	Payer	5 years	Markov cohort model	RCTs (Systematic review)	VAS converted from ACR20, ACR50, ACR70 and no ACR responses	3%
Schipper et al. 2011, the Netherland [30]	Early RA, no previous cDMARDs	1.)1.TNFi+MTX ➔ 2.TNFi+MTX➔ RTX+MTX 2.)MTX+LEF➔ 1.TNFi+MTX ➔ 2.TNFi+MTX➔ RTX+MTX 3.)MTX(MTX+LEF ➔ 1.TNFi+MTX ➔ 2.TNFi+MTX➔ RTX+MTX	Comparison of treatment 3 strategies containing TNFi	Payer, societal	5 years	Patient-level Markov model	Registries (Nijmegen and DREAM)	EQ-5D	4%
Soini et al. 2012, Finland [20]	Moderate to severe RA, inadequate response to at least 1 cDMARD	TOC+MTX / ADA+MTX / ETN+MTX ➔ RTX+MTX ➔ IFX+MTX ➔ LEF ➔ CSA ➔ MTX	MTX➔ RTX+MTX ➔ IFX+MTX ➔ LEF ➔ CSA ➔ MTX	Payer, societal	Lifetime	Individual sampling model	MTC	EQ-5D converted from HAQ, QoL = 0.82–0.11 x HAQ—0.07 x HAQ²	3%
Spalding & Hay 2006, USA [14]	Early RA (< 3 months), no previous cDMARDs	ADA+MTX or ADA or IFX+MTX or ETN	MTX	Payer, societal	Lifetime	Markov model	Several RCTs	HUI3 converted from HAQ, QoL = 0.76–0.28 x HAQ + 0.05 x FEMALE + 0,001 x AGE	3%
Tanno et al. 2006, Japan [35]	RA, inadequate response to busillamine (cDMARD)	ETN ➔ cDMARDs	cDMARDs: MTX ➔ SSZ ➔ MTX+SSZ ➔ no cDMARD	Societal	Lifetime	Markov model	RCT	EQ-5D converted from HAQ, QoL = 0.74–0.17 x HAQ	Costs 6%, benefits 1.5%
Wailoo et al. 2008, USA [40]	Established RA	ADA / IFX / ETN / ANA ➔ cDMARD	Comparison of biologics	Payer (Medicare)	Lifetime	Model, unspecified	Meta-analysis	EQ-5D converted from HAQ	3%
van den Hout et al. 2009, the Netherlands [19]	Early RA (≤ 2 years), no previous cDMARDs	1.)MTX ➔ MTX+SSZ ➔ MTX+SSZ+HCQ➔ MTX+SSZ+HCQ+CS ➔ IFX+MTX ➔ MTX+CSA+CS ➔ LEF ➔ AZA+CS 2.)IFX+MTX➔ SSZ ➔ LEF ➔MTX+CSA+ CS ➔ GST+CS ➔ AZA+CS 3.)MTX(SSZ➔ LEF ➔ IFX+MTX ➔ GST+ CS ➔ MTX+CSA+CS ➔ AZA+ CS 4.)MTX+SSZ+CS➔ MTX+CSA+ CS ➔ IFX+MTX ➔ LEF ➔ GST+ CS ➔ AZA+CS	Comparison of treatment 4 strategies containing TNFi	Societal	2 years	Empiric CUA, no modelling used10	RCT	EQ-5D (British and Dutch valuations), SF6D, TTO	3%
Welsing et al. 2004, the Netherland [23]	Active RA, inadequate response to at least 2 cDMARDs (SSZ, MTX)	ETN➔Usual care or LEF➔ETN(Usual care or ETN➔LEF(Usual care	Usual care or LEF(Usual care	Societal, Payer (Third party payer)	5 years	Markov model	RCTs	EQ-5D converted from DAS28 responses	4%
Vera-Llonch et al. 2008a, USA [17]	Moderate to severe RA, inadequate response to MTX	ABT+MTX	MTX	Third party payer	10 years, lifetime	Patient-level simulation model	RCT	EQ-5D converted from HAQ	3%
Vera-Llonch et al. 2008b, USA [43]	Moderate to severe RA, inadequate response to TNFi	ABT+MTX	MTX	Third party payer	10 years, lifetime	Patient-level simulation model	RCT	EQ-5D converted from HAQ	3%
Wong et al. 2002 [39]	Active refractory RA	IFX+MTX	MTX	Payer, societal	Lifetime (model), 54 weeks (treatment)	Markov cohort model	RCT	VAS	3%
Wu et al. 2012, China [29]	Moderate to severe RA, inadequate response to at least 2 cDMARDs (including MTX)	ETN / IFX / ADA➔ cDMARDs or ETN(RTX➔ cDMARDs or IFX(RTX➔ cDMARDs or ADA(RTX➔ cDMARDs	cDMARDs: GST ➔ LEF ➔ CSA ➔ MTX	Payer, societal	Lifetime	Markov cohort model	RCTs	HUI-3 converted from HAQ, QoL = 0.76–0.28 x HAQ + 0.05 x FEMALE	3%
Yuan et al. 2010, USA [42]	Active RA, inadequate response to a TNFi	ABA+MTX / RTX+MTX ➔ MTX	MTX	Payer	Lifetime	Patient-level simulation model	RCTs	EQ-5D converted from HAQ	3%

➔ = switch to next treatment in case of an inadequate response, ABT = abatacept, ADA = adalimumab, ANA = anakinra, AZA = azathioprine, bDMARD = biologic disease-modifying antirheumatic drugs, CADTH = Canadian Agency for Drugs and Technologies in Health, cDMARD = conventional disease-modifying antirheumatic drugs, CER = certolizumab pegol, CS = corticosteroids, CSA = cyclosporin A, DAS28 = Disease Activity Score 28, D-PEN = D-Penicillin, EQ-5D = EuroQol-5D, ETN = etanercept, GOL = golimumab, GST = Gold, HAQ = Health Assessment Questionnaire, HCQ = hydroxychloroquine, HUI-2 = Health Utility Index 2, HUI-3 = Health utility Index 3, ICER = Incremental cost-effectiveness ratio, IFX = infliximab, LEF = leflunomide, MTC = mixed-treatment comparison, MTX = methotrexate, NSAID = non-steroidal anti-inflammatory drug, QALY = quality-adjusted life year, QoL = quality of life, RA = rheumatoid arthritis, RCT = randomized controlled trial, RTX = rituximab, SF-6D = Short Form 6D, SSZ = sulfasalazine, TNFi = TNF inhibitor, TOC = tocilizumab, TTO = Time Trade-off, UK NHS = The National Health Service of the United Kingdom, VAS = the Visual Analogue Scale,

*People and society tend to value present costs and benefits more than future ones. This is taken into account by discounting future costs and benefits with a predefined rate.

### Cost-effectiveness of biologics in patients with early RA and naïve to cDMARDs

The cost-effectiveness of biologics for patients with early RA and naïve to cDMARDs were analysed in seven studies (Table 2). Four studies performed a comparison between biologics and cDMARDs [14,21,28,38]. The ICERs of TNFi in comparison to cDMARDs ranged from 39,000 to 1 273,000 €/QALY when only direct costs were considered (Table 2). IFX was associated with the highest ICERs ranging from 422,000 to 1 273,000 €/QALY while ICERs for ETN and ADA as a monotherapy were below 100,000 €/QALY. As a combination therapy with MTX, ICERs for ETN and ADA were substantially higher. If both direct and indirect costs were considered, ICERs for biologics were slightly more favourable.

**Table 2:** Cost-effectiveness of biologics in cDMARD naïve patients.

Treatments	Study	ICER €/QALY (only direct costs)	ICER €/QALY (direct and indirect costs)	Results of deterministic sensitivity analysis €/QALY	Source of research funding
**TNFi vs. cDMARDs**					
IFX	Chen et al. 2006 [28]	1 273,007	-	40,876—dominated	NICE (UK)
	Davies et al. 2009 [21]	Extended dominance by ADA	Extended dominance by ADA	-	Abbott
	Spalding & Hay 2006 [14]	422,215	-	422,114–573,650	University of Southern California
ADA	Chen et al. 2006 [28]	152,021 (ADA+MTX)	-	40,876—dominated (ADA+MTX)	NICE (UK)
	Chen et al. 2006 [28]	58,672 (ADA)	-	36,983—dominated (ADA)	NICE (UK)
	Davies et al. 2009 [21]	41,178 (ADA+MTX)	20,413	31,435–61,124	Abbott
	Davies et al. 2009 [21]	37,309 (ADA+MTX ➔ ETN)	-	-	Abbott
	Spalding & Hay 2006 [14]	200,620 (ADA+MTX)	-	200,570 (ADA+MTX)	University of Southern California
	Spalding & Hay 2006 [14]	65,745 (ADA)	-	67,962 (ADA)	University of Southern California
ETN	Spalding & Hay 2006 [14]	92,503	81,408	80,027–108,051	University of Southern California
	Davies et al. 2009 [21]	Extended dominance by ADA	Extended dominance by ADA	-	Abbott
	Kobelt et al. 2011 [38]	38,639	15,315	2,473–38,639	Wyeth (now Pfizer)
	Chen et al. 2006 [28]	332,850 (ETN+MTX)	-	35,037—dominated (ETN+MTX)	NICE (UK)
	Chen et al. 2006 [28]	96,157 (ETN)	-	35,037–231,633 (ETN)	NICE (UK)
**Comparison of treatment strategies containing TNFi**					
**1.)**MTX➔MTX+SSZ➔ MTX+SSZ+HCQ➔ MTX+SSZ+HCQ+CS ➔IFX(MTX+CSA+ CS ➔ LEF➔AZA+CS 2.)IFX(SSZ➔LEF➔ MTX+CSA+CS➔GST+CS➔AZA+CS	Van den Hout et al. 2009 [19]	2 vs.1: 215,256	2 vs.1: 147,280	24,924–362,537	Dutch Health Care Insurance Board, Schering-Plough and Centocor (now Janssen Biologics B.V)
**1.)**1.TNFi➔2.TNFi➔RTX **2.)**MTX+LEF➔1.TNFi➔ 2.TNFi➔RTX **3.)**MTX➔MTX+LEF ➔1.TNFi ➔2.TNFi➔RTX	Schipper et al. 2011 [30]	2 vs.3: 462,576	2 vs.3: 461,476	2 vs.1: 456,946–791,788	Wyeth (now Pfizer)
	Schipper et al. 2011 [30]	1 vs.3: 145,784	1 vs.3: 143,831	1 vs.3: 120,136–545,603	Wyeth (now Pfizer)
	Schipper et al. 2011 [30]	2 vs.1: 1 dominates	2 vs.1: 1 dominates	-	Wyeth (now Pfizer)
**1.)**cDMARDs ➔ 1.TNFi ➔ 2.TNFi ➔ 3.TNFi **2.**➔1.TNFi ➔ 2.TNFi ➔ 3.TNFi ➔ cDMARDs **3.)**NSAID ➔ cDMARDs➔ 1.TNFi ➔2.TNFi➔3.TNFi	Finckh et al. 2009 [27]	1 vs.3: 4,234	1 vs.3: 1 is cost-saving	1 vs.3: 1 is cost saving—14,738	Arthritis research foundation and an anonymous donor
	Finckh et al. 2009 [27]	2 vs.3: 635,597	2 vs.3: 471,575	2 vs.3: 30,624–3 dominates	Arthritis research foundation and an anonymous donor
	Finckh et al. 2009 [27]	2 vs.1: 1 dominates	2 vs.1: 1 dominates	2 vs.1: 40,956–1 dominates.	Arthritis research foundation and an anonymous donor

➔ = switch to next treatment in case of an inadequate response, ADA = adalimumab, AZA = azathioprine, cDMARD = conventional disease-modifying antirheumatic drugs, CS = corticosteroids, CSA = cyclosporin A, ETN = etanercept, GST = Gold, HCQ = hydroxychloroquine, ICER = Incremental cost-effectiveness ratio, IFX = infliximab, LEF = leflunomide, MTX = methotrexate, NICE = National Institute for Health and Care Excellence, NSAID = non-steroidal anti-inflammatory drug, QALY = quality-adjusted life year, SSZ = sulfasalazine, TNFi = TNF inhibitor

Three out of the seven studies examined the cost-effectiveness of different treatment strategies for early RA including TNFi in all treatment options, with only its time of usage in a treatment sequence being altered [19,27,30]. Two studies found a late introduction of TNFi to be a dominant strategy compared to initiation of the treatment with TNFi. Meanwhile van den Hout and colleagues found the ICER for TNFi as a first-line treatment option to be 215,000 €/QALY compared to its later introduction (Table 2).

### Cost-effectiveness of biologics among patients with an inadequate response to cDMARD

There were 21 studies comparing the biologics and cDMARDs in patients with an insufficient response to cDMARDs (Table 3). When only direct costs were considered ICERs for IFX, ADA and ETN were 12,000–282,000; 44,000–274,000 and 40,000–708,000, respectively. ABT and TOC were associated with narrower ranges of ICERs (42,000 to 47,000 and 19,000 to 21,000, respectively). ICERs below 35,000 €/QALY were found in three studies [20,29,51] and below 50,000 €/QALY in ten studies [15,17,28,39,41,49,52]. The quality scores of the studies were not associated with the magnitude of ICER values. Adequate comparator was applied in nine of 21 CUAs [15,23,28,29,32,35,36,46,50]. These studies provided higher ICERs compared to other studies: only one CUAs with an adequate comparison treatment provided ICERs below 35,000 €/QALY for biologics when considering only direct costs [29].

**Table 3:** Cost-effectiveness of biologics in comparison with cDMARD among patients with an insufficient response to cDMARD.

Biologic	Study	ICER €/QALY (only direct costs)	ICER €/QALY (direct and indirect costs)	Results of deterministic sensitivity analysis €/QALY	Source of research funding
**IFX**	Bansback et al. 2005 [41]	69,717–93,665	-	-	Abbott
	Barbieri et al. 2005 [51]	12,438–89,108	-	9,325–103,753	Schering-Plough
	Barton et al. 2004 [50]	166,921	-	96,287–213,008	NICE (UK)
	CADTH 2010 [48]	Extended dominance by ADA	-	-	Health Canada and the governments of provinces and territories
	Chen et al. 2006 [28]	59,173–270,563 (IFX➔cDMARDs)	-	37,957—dominated (IFX➔cDMARDs)	NICE (UK)
	Chen et al. 2006 [28]	73,772 (cDMARDs➔IFX)	-	50,027–117,763 (cDMARDs➔IFX)	NICE (UK)
	Coyle et al. 2006 [46]	98,132 (IFX➔GST)	-	85,279–138,948 (IFX➔GST)	Health Canada and the governments of provinces and territories
	Coyle et al. 2006 [46]	84,931 (GST➔IFX)	-	71,298–101,084 (GST➔IFX)	Health Canada and the governments of provinces and territories
	Jobanputra et al. 2002 [32]	282,151 (IFX➔cDMARDs)	-	128,590–641,955 (IFX➔cDMARDs)	NICE (UK)
	Jobanputra et al. 2002 [32]	230,698 (cDMARDs➔IFX)	-	68,157–413,593 (cDMARDs➔IFX)	NICE (UK)
	Kobelt et al. 2003 [52]	38,945–76,392	4,684–65,635	IFX is cost saving—60,597	Schering-Plough
	Lekander et al. 2010 [26]	-	27,321	10,005–56,246	Schering-Plough
	Marra et al. 2007 [44]	-	30,267–66,008	IFX dominates—139,343	Canadian Arthritis Network
	Wu et al. 2012 [29]	20,254 (IFX)	20,150 (IFX)	-	Shanghai Hospital Association, National Natural Science Foundation of China and Shanghai Natural Science Foundation
	Wu et al. 2012 [29]	21,946 (IFX➔RTX)	21,833 (IFX➔RTX)	-	Shanghai Hospital Association, National Natural Science Foundation of China and Shanghai Natural Science Foundation
	Wong et al. 2002 [39]	44,737	13,348	IFX is cost saving—137,292	Schering-Plough and National Institutes of Health
**ADA**	Bansback et al. 2005 [41]	49,284–63,493 (ADA+MTX)	-	-	Abbott
	Bansback et al. 2005 [41]	59,949–94,478 (ADA)	-	-	Abbott
	CADTH 2010 [48]	92,326	-	-	Health Canada and the governments of provinces and territories
	Chen et al. 2006 [28]	58,784–125,354 (ADA+MTX➔ cDMARDs)	-	37,178–291,974 (ADA+MTX➔ cDMARDs)	NICE (UK)
	Chen et al. 2006 [28]	67,349–274,456 (ADA➔ cDMARDs)	-	41,266- dominated (ADA➔ cDMARDs)	NICE (UK)
	Chen et al. 2006 [28]	57,811 (cDMARDs➔ ADA+MTX)	-	43,018–83,699 (cDMARDs➔ ADA+MTX)	NICE (UK)
	Chen et al. 2006 [28]	78,054 (cDMARDs➔ADA)	-	52,750–124,770 (cDMARDs➔ADA)	NICE (UK)
	Wu et al. 2012 [29]	43,943 (ADA)	43,876 (ADA)	-	Shanghai Hospital Association, National Natural Science Foundation of China and Shanghai Natural Science Foundation
	Wu et al. 2012 [29]	38,689 (ADA➔ RTX)	38,641 (ADA➔ RTX)	-	Shanghai Hospital Association, National Natural Science Foundation of China and Shanghai Natural Science Foundation
**ETN**	Bansback et al. 2005 [41]	51,581–74,972 (ETN+MTX)	-	-	Abbott
	Bansback et al. 2005 [41]	53,265–61,274 (ETN)	-	-	Abbott
	Barton et al. 2004 [50]	122,754	-	73,350–157,370	NICE (UK)
	Brennan et al. 2004 [49]	39,740	18,950	18 950–103 145	Not stated, two of authors are employees of Wyeth (now Pfizer)
	CADTH 2010 [48]	Dominated by ADA	-	-	Health Canada and the governments of provinces and territories
	Chen et al. 2006 [28]	55,475–96,935 (ETN+MTX➔ cDMARDs)	-	34,648–187,058 (ETN+MTX➔ cDMARDs)	NICE (UK)
	Chen et al. 2006 [28]	59,173–92,264 (ETN➔cDMARDs)	-	36,399–185,695 (ETN➔ cDMARDs)	NICE (UK)
	Chen et al. 2006 [28]	46,327 (cDMARDs➔ ETN+MTX)	-	35,037–66,181 (cDMARDs➔ ETN+MTX)	NICE (UK)
	Chen et al. 2006 [28]	46,132 (cDMARDs➔ ETN)	-	35,232–65,013 (cDMARDs➔ ETN)	NICE (UK)
	Coyle et al. 2006 [46]	125,661 (ETN➔ GST)	-	109,335–173,251 (ETN➔ GST)	Health Canada and the governments of provinces and territories
	Coyle et al. 2006 [46]	109,161 (GST➔ETN)	-	94 919–129,916 (GST(ETN)	Health Canada and the governments of provinces and territories
	Jobanputra et al. 2002 [32]	202,218 (ETN➔ cDMARDs)	-	93,643–448,885 (ETN➔ cDMARDs)	NICE (UK)
	Jobanputra et al. 2002 [32]	174,388 (cDMARDs➔ ETN)	-	51,662–312,186 (cDMARDs➔ ETN)	NICE (UK)
	Kobelt et al. 2005 [36]	69,550 (ETN+MTX)	49,314–72,058 (ETN+MTX)	33,704–69,550	Wyeth (now Pfizer)
	Kobelt et al. 2005 [36]	-	Dominated by ETN+MTX (ETN)	-	Wyeth (now Pfizer)
	Lekander et al. 2013 [25]	-	52,671 (ETN+cDMARD)	33,922–78,770 (ETN+cDMARD)	Wyeth (now Pfizer)
	Lekander et al. 2013 [25]	-	68,535 (ETN)	40,818–127,988 (ETN)	Wyeth (now Pfizer)
	Soini et al. 2012 [20]	22,745	-	9,437–57,025	Roche
	Tanno et al. 2006 [35]	-	25,993	19,547–32,439	Ministry of Education, Science, Sports and Culture and the Ministry of Health, Japan
	Welsing et al. 2004 [23]	233,867 (LEF➔ ETN➔ Usual care vs. Usual care)	216,059 (LEF➔ ETN➔ Usual care vs. Usual care)	-	Not stated
	Welsing et al. 2004 [23]	413,169 (ETN➔ LEF➔ Usual care vs. Usual care)	392,539 (ETN➔ LEF➔Usual care vs. Usual care)	-	Not stated
	Welsing et al. 2004 [23]	440,322 (LEF➔ ETN(Usual care vs. LEF➔ Usual care)	419,588 (LEF➔ ETN➔ Usual care vs. LEF➔ Usual care)	-	Not stated
	Welsing et al. 2004 [23]	708,060 (ETN➔ LEF➔ Usual care vs. LEF➔ Usual care)	683,041 (ETN➔ LEF➔ Usual care vs. LEF➔ Usual care)	-	Not stated
	Wu et al. 2012 [29]	58,711 (ETN)	58,684 (ETN)	-	Shanghai Hospital Association, National Natural Science Foundation of China and Shanghai Natural Science Foundation
	Wu et al. 2012 [29]	50,409 (ETN➔ RTX)	50,389 (ETN➔ RTX)	-	Shanghai Hospital Association, National Natural Science Foundation of China and Shanghai Natural Science Foundation
**ABT**	CADTH 2010 [48]	Extended dominance by ADA	-	-	Health Canada and the governments of provinces and territories
	Vera-Llonch et al. 2008a [17]	42,382–47,177	-	36,976–69,134	Bristol-Myers Squibb
**GOL**	CADTH 2010 [48]	Extended dominance by ADA	-	-	Health Canada and the governments of provinces and territories
**TOC**	Soini et al. 2012 [20]	18,693–20,776	18,731–20,813	7,629–53,17	Roche
**TNFi**	Brennan et al. 2007 [15]	46,486 (TNFi as a group)	-	24,378–93,833	The British Society for Rheumatology (BSR)
	Kobelt et al. 2004 [16]	62,419	61,016	51,759–180,244	Österlund and Kock Foundations, The King Gustav V 80 year fund and The Reumatikerförbundet
	Lekander et al. 2013 [25]	75,799 (TNFi+cDMARD)	57,092 (TNFi+cDMARD)	34,472–88,294 (TNFi+cDMARD)	Wyeth (now Pfizer)
	Lekander et al. 2013 [25]	106,062 (TNFi)	88,146 (TNFi)	50 315–169 383 (TNFi)	Wyeth (now Pfizer)

➔ = switch to next treatment in case of an inadequate response, ABT = abatacept, ADA = adalimumab, CADTH = Canadian Agency for Drugs and Technologies in Health, cDMARD = conventional disease-modifying antirheumatic drugs, CER = certolizumab pegol, ETN = etanercept, GOL = golimumab, GST = Gold, ICER = Incremental cost-effectiveness ratio, IFX = infliximab, LEF = leflunomide, MTX = methotrexate, NICE = National Institute for Health and Care Excellence, QALY = quality-adjusted life year, SSZ = sulfasalazine, TNFi = TNF inhibitor, TOC = tocilizumab

Six studies performed comparisons between different biologics used in patients with an inadequate response to cDMARDs [20,24,28,32,33,50]. The results of these studies were contradictory. Two studies [20,24] found ETN to be dominant over IFX and ADA, while three of the other studies[28,32,50] reported ICERs ranging from 23,000 to 109,000 €/QALY for ETN when only direct costs were included (Table 4). Two studies comparing TOC and ETN found TOC to be the dominant strategy. None of these CUAs included indirect costs.

**Table 4:** Comparison of biologics in patients with an insufficient response to cDMARD.

Biologic	Comparator	Study	ICER €/QALY (only direct costs)	Results of deterministic sensitivity analysis €/QALY	Source of research funding
**IFX**	ETN	Nguyen et al. 2012 [24]	ETN dominates	-	One of the authors was funded by UCB Pharma
	CER	Nguyen et al. 2012 [24]	CER dominates	-	One of the authors was funded by UCB Pharma
**ADA**	GOL	Nguyen et al. 2012 [24]	ADA dominates	-	One of the authors was funded by UCB Pharma
	ETN	Nguyen et al. 2012 [24]	ETN dominates	-	One of the authors was funded by UCB Pharma
	IFX	Chen et al. 2006 [28]	4,983—IFX is cost saving (ADA➔ cDMARDs)	-	NICE (UK)
	IFX	Chen et al. 2006 [28]	ADA dominates (cDMARDs➔ ADA)	-	NICE (UK)
**ETN**	IFX	Barton et al. 2004 [50]	68,373	42,760–88,266	NICE (UK)
	IFX	Jobanputra et al. 2002 [32]	109,297 (ETN➔ cDMARDs)	51,908–231,484 (ETN➔ cDMARDs)	NICE (UK)
	IFX	Jobanputra et al. 2002 [32]	101,714 (cDMARDs➔ETN)	30,597–180,270 (cDMARDs➔ ETN)	NICE (UK)
	IFX	Chen et al. 2006 [28]	38,541–47,884 (ETN➔ cDMARDs)	-	NICE (UK)
	IFX	Chen et al. 2006 [28]	23,553 (cDMARDs➔ ETN)	-	NICE (UK)
	ADA	Soini et al. 2012 [20]	ETN dominates	-	Roche
	ADA	Chen et al. 2006 [28]	35,621–61,315 (ETN➔ cDMARDs)	-	NICE (UK)
	ADA	Chen et al. 2006 [28]	22,579–30,755 (cDMARDs➔ ETN)	-	NICE (UK)
**GOL**	ETN	Nguyen et al. 2012 [24]	ETN dominates	-	One of the authors was funded by UCB Pharma
	CER	Nguyen et al. 2012 [24]	CER dominates	-	One of the authors was funded by UCB Pharma
**CER**	ETN	Nguyen et al. 2012 [24]	1 756,213	-	One of the authors was funded by UCB Pharma
**TOC**	ETN	Diamantopoulos et al. 2012 [33]	TOC dominates	TOC dominates—19,187	Roche
	ETN	Soini et al. 2012 [20]	TOC dominates—6,673	-	Roche

➔ = switch to next treatment in case of an inadequate response, ADA = adalimumab, CER = certolizumab pegol, ETN = etanercept, GOL = golimumab, ICER = Incremental cost-effectiveness ratio, IFX = infliximab, NICE = National Institute for Health and Care Excellence, QALY = quality-adjusted life year, TOC = tocilizumab

### Cost-effectiveness of biologics among patients with an inadequate response to at least one TNF inhibitor

Eight CUAs compared biologics and cDMARDs in patients who had had an insufficient response to at least one TNFi [22,25,31,34,42,43,53,54]. RTX was associated with the lowest ICERs ranging from 26,000 to 48,000 €/QALY (Table 5). Three of four studies evaluating RTX provided ICERs below 35,000 €/QALY and none of the studies reported ICERs more than 50,000 €/QALY. ANA was associated with the highest ICERs with a range of 234,000–1 347,000 €/QALY. ICERs for the other agents ranged from 41,000 to 143,000 €/QALY. Inadequate comparator (MTX) was applied in three studies [34,42,43], and one study [25] did not specify the comparator cDMARDs. However, the ICERs of these studies did not differ from those of the other studies. Results of the four studies comparing one biologic to another [18,31,34,53] indicated RTX as the most cost-effective biologic among patients with an insufficient response to a TNFi (Table 6).

**Table 5:** Cost-effectiveness of biologics in comparison with cDMARD among patients with an insufficient response to at least one TNF inhibitor.

Biologic	Study	ICER €/QALY (only direct costs)	ICER €/QALY (direct and indirect costs)	Results of deterministic sensitivity analysis €/QALY	Source of research funding
**RTX**	Yuan et al. 2010 [42]	47,931	-	57,370–96,012	BMS
	Kielhorn et al. 2008 [31]	28,594	-	9,758–67,321	Roche
	Brodszky et al. 2010 [34]	26,304–46,389	31,382–37,266	-	Center for Public Affairs Studies Foundation and Roche
	Hallinen et al. 2010 [54]	34,269	-	24,929–52,929	Roche
	Malottki et al. 2011 [53]	30,021	-	16,220–65,448	NICE (UK)
**IFX**	Hallinen et al. 2010 [54]	40,923	-	36,174–48,483	Roche
	Malottki et al. 2011 [53]	51,362	-	40,976–98,029	NICE (UK)
**ADA**	Hallinen et al. 2010 [54]	57,713	-	48,963–68,930	Roche
	Malottki et al. 2011 [53]	48,801	-	39,980–87,216	NICE (UK)
**ETN**	Hallinen et al. 2010 [54]	57,068	-	48,294–68,285	Roche
	Malottki et al. 2011 [53]	55,346	-	44,248–108,558	NICE (UK)
	Lekander et al. 2013 [25]	-	74,743 (ETN+cDMARD)	47,164–113,453 (ETN+DMARD)	Wyeth (now Pfizer)
	Lekander et al. 2013 [25]	-	88,861 (ETN)	53,769–175,126 (ETN)	Wyeth (now Pfizer)
**ABT**	Hallinen et al. 2010 [54]	75,910	-	65,232–90,234	Roche
	Malottki et al. 2011 [53]	54,635	-	45,671–90,062	NICE (UK)
	Vera-Llonch et al. 2008b [43]	45,275–49,802	-	40,211–79,438	Not stated, One of authors was an employee of BMS
	Yuan et al. 2010 [42]	41,207	-	49,912–81,509	BMS
**ANA**	Clark et al. 2004 [22]	620,109–1 347,287 (ANA➔cDMARDs)	-	100,378–671,413	NICE (UK)
	Clark et al. 2004 [22]	234,214–292,210 (cDMARDs➔ANA)	-	82,533–216,370	NICE (UK)
**TNFi**	Lekander et al. 2013 [25]	101,618 (TNFi+cDMARD)	84,363 (TNFi+cDMARD)	50,316–134,016 (TNFi+cDMARD)	Wyeth (now Pfizer)
	Lekander et al. 2013 [25]	143,745 (TNFi)	126,813 (TNFi)	71,022–328,903 (TNFi)	Wyeth (now Pfizer)

➔ = switch to next treatment in case of an inadequate response, ABT = abatacept, ADA = adalimumab, ANA = Anakinra, BMS = Bristol-Myers Squibb, cDMARD = conventional disease-modifying antirheumatic drugs, ETN = etanercept, ICER = Incremental cost-effectiveness ratio, IFX = infliximab, NICE = National Institute for Health and Care Excellence, QALY = quality-adjusted life year, RTX = rituximab, TNFi = TNF inhibitor

**Table 6:** Comparison of biologics among patients with an insufficient response to at least one TNF inhibitor.

Biologic	Comparator	Study	ICER €/QALY (only direct costs)	ICER €/QALY (direct and indirect costs)	Results of deterministic sensitivity analysis €/QALY	Source of research funding
**RTX**	Another TNFi	Brodszky et al. 2010 [34]	RTX dominates	RTX dominates	-	Center for Public Affairs Studies Foundation and Roche
	2.TNFi➔ 3.TNFi	Lindgren et al. 2009 [45]	RTX dominant	RTX dominant	RTX dominates—41,044	Roche
	ADA ➔ IFX ➔ cDMARDs	Merkesdal et al. 2010 [18]	27,776	17,634	8,050–54,441	Roche
	ADA ➔ IFX ➔ cDMARDs	Kielhorn et al. 2008 [31]	22,581	-	-	Roche
**IFX**	RTX	Malottki et al. 2011 [53]	RTX dominates	-	5,833—RTX dominates	NICE (UK)
**ADA**	RTX	Malottki et al. 2011 [53]	RTX dominates	-	612—RTX dominates	NICE (UK)
	ETN	Malottki et al. 2011 [53]	ADA dominates	-	ADA dominates-103,578	NICE (UK)
	IFX	Malottki et al. 2011 [53]	ADA dominates	-	27,033–40,834	NICE (UK)
**ETN**	RTX	Malottki et al. 2011 [53]	RTX dominates	-	RTX dominates	NICE (UK)
	IFX	Malottki et al. 2011 [53]	649,782	-	55,915—IFX dominates	NICE (UK)
**ABT**	RTX	Malottki et al. 2011 [53]	185,815	-	73,273–1 225,153	NICE (UK)
	ADA	Malottki et al. 2011 [53]	66,017	-	57,053–119,656	NICE (UK)
	ETN	Malottki et al. 2011 [53]	53,781	-	47,663–71,992	NICE (UK)
	IFX	Malottki et al. 2011 [53]	59,329	-	52,500–81,952	NICE (UK)

➔ = switch to next treatment in case of an inadequate response, ABT = abatacept, ADA = adalimumab, ETN = etanercept, ICER = Incremental cost-effectiveness ratio, IFX = infliximab, NICE = National Institute for Health and Care Excellence, QALY = quality-adjusted life year, RTX = rituximab, TNFi = TNF inhibitor

### Other studies

Three studies did not specify patients’ previous treatments, and therefore were not included in the subgroups described above [37,40,47]. Farahani *et al*. estimated ICER for ETN in comparison to cDMARDs to be 71,000 €/QALY while applying the efficacy estimates based on a RCT and 150,000 €/QALY when effectiveness estimates from an observational study were used [37]. Chiou *et al*. and Wailoo *et al*. performed comparisons of different biologics [40,47]. Both studies reported ETN to be dominant over IFX. Chiou *et al*. also found ETN to dominate ADA while Wailoo *et al*. estimated ICER of 95,000 €/QALY for ETN in comparison to ADA.

### Quality of the included studies

The average quality scores of the 41 studies included in the present review were 25.7 out of 35 (range 17 to 31) and 32.3 out of 57 (range 16 to 46) when evaluated using BMJ checklist and Philips’ list, respectively (Table 7). The corresponding average percentages of the applicable items fulfilled were 81 (range 57 to 100) and 62 (range 31 to 90) for BMJ check list and Philips’ list, respectively. The most frequent quality issues were the incomplete reporting of the data sources, inappropriate comparator treatments, defects in the sensitivity analysis and the lack of quality assessment of data used.

**Table 7:** Results of quality assessment.

Study	BMJ quality scores, max = 35 (items applicable in each study)	Applicable items %	Philip’s quality scores, max = 57 (items applicable in each study)	Applicable items %	Quality category
Bansback et al. 2005, Sweden [41]	23 (31)	74	38 (52)	73	Adequate
Barbieri et al. 2005, UK [51]	25 (31)	81	23 (53)	43	Poor
Barton et al. 2004, UK [50]	29 (31)	94	40 (49)	82	Good
Brennan et al. 2004, UK [49]	29 (33)	88	30 (54)	56	Adequate
Brennan et al. 2007, UK [15]	26 (32)	81	37 (49)	76	Good
Brodszky et al. 2010, Hungary [34]	19 (30)	63	16 (52)	31	Poor
CADTH 2010, Canada [48]	17 (30)	57	18 (53)	34	Poor
Chen et al. 2006, UK [28]	31 (31)	100	46 (51)	90	Good
Chiou et al. 2004, [47]	23 (31)	74	20 (53)	38	Poor
Clark et al. 2004, UK [22]	30 (31)	97	40 (50)	80	Good
Coyle et al. 2006, Canada [46]	29 (31)	94	28 (52)	54	Adequate
Davies et al. 2009 USA [21]	29 (32)	91	38 (55)	69	Good
Diamantopoulos et al. 2012, Italy [33]	25 (32)	78	32 (55)	58	Adequate
Farahani et al. 2006, Canada [37]	19 (27)	70	No modelling used	-	Poor
Finckh et al. 2009, USA [27]	28 (32)	88	36 (54)	67	Good
Hallinen et al. 2010, Finland [54]	29 (31)	94	28 (52)	54	Adequate
Jobanputra et al. 2002, UK [32]	28 (31)	90	34 (49)	69	Good
Kielhorn et al. 2008, UK [31]	25 (31)	81	37 (53)	70	Adequate
Kobelt et al. 2003, UK & Sweden [52]	23 (32)	72	22 (49)	45	Poor
Kobelt et al. 2004, Sweden [16]	25 (30)	83	No modelling used	-	Adequate
Kobelt et al. 2005, Sweden [36]	22 (33)	67	28 (53)	53	Poor
Kobelt et al. 2011, Sweden [38]	26 (33)	79	28 (54)	52	Poor
Lekander et al. 2010, Sweden [26]	23 (33)	70	31 (51)	61	Poor
Lekander et al. 2013, Sweden [25]	24 (33)	73	37 (53)	70	Adequate
Lindgren et al. 2009, Sweden [45]	22 (33)	67	34 (48)	71	Adequate
Malottki et al. 2011, UK [53]	29 (31)	94	46 (52)	88	Good
Marra et al. 2007, Canada [44]	27 (33)	82	31 (54)	57	Adequate
Merkesdal et al. 2010, Germany [18]	27 (32)	84	34 (52)	65	Adequate
Nguyen et al. 2012, USA [24]	25 (31)	81	28 (55)	51	Poor
Schipper et al. 2011, the Netherlands [30]	25 (33)	76	34 (52)	65	Adequate
Soini et al. 2012, Finland [20]	31 (33)	94	42 (54)	78	Good
Spalding & Hay 2006, USA [14]	23 (32)	72	30 (52)	58	Poor
Tanno et al. 2006, Japan [35]	29 (32)	91	23 (51)	45	Adequate
Wailoo et al. 2008, USA [40]	25 (31)	81	36 (55)	65	Adequate
van den Hout et al. 2009, the Netherlands [19]	29 (31)	94	No modelling used	-	Good
Welsing et al. 2004, the Netherlands [23]	22 (32)	69	27 (55)	49	Poor
Vera-Llonch et al. 2008a, USA [17]	26 (32)	81	37 (50)	74	Good
Vera-Llonch et al. 2008b, USA [43]	27 (32)	84	37 (50)	74	Good
Wong et al. 2002 [39]	23 (33)	70	24 (51)	47	Poor
Wu et al. 2012, China [29]	30 (32)	94	41 (54)	76	Good
Yuan et al. 2010, USA [42]	23(32)	72	36 (53)	68	Adequate

## Discussion

We performed a systematic literature review of cost-effectiveness of biologics used for the treatment of RA. After the literature search and the selection process of the initially identified reports, 41 original articles were included in the current review. While considering only direct costs, the ICERs of the TNFis ranged from 39,000 to 1 273,000 €/ QALY in comparison to cDMARD in patients naïve to cDMARDs. Among patients with an inadequate response to cDMARDs, biologics were associated with ICERs ranging from 12,000 to 708,000 €/QALY. In this setting, none of the biologics appeared to be more cost-effective than any of the others. ICERs for the second line biologics ranged from 26,000 to 1 347,000 €/QALY in comparison to cDMARDs among patients with an inadequate response to TNFi. In this patient subgroup RTX was the most and ANA the least cost-effective biologic. The quality assessment revealed several problems, namely insufficient reporting of data sources and problematic methodological details, which possibly reduce the validity of the results.

When assessing whether biologics are cost-effective or not, it should be known what the willingness to pay for an additional QALY is. There is no widely accepted WTP threshold value for ICER although the National Institute for Health and Care Excellence (NICE) has published a threshold of 20,000–30,000 £/QALY (~24,000–35,000 €/QALY) in United Kingdom [55]. Based on this statement by NICE we used the WTP threshold of 35,000 €/QALY. With this threshold biologics are not cost-effective in cDMARD naïve patients. However, also much higher WTP thresholds have been proposed and applied in the literature, but even with the 100,000 €/QALY threshold biologics do not seem to be cost-effective in this patient subgroup. Slightly more preferable ICERs for ADA and ETN monotherapies do not count either: TNFi monotherapy has later been found less effective than its combination with MTX and therefore, biologics as monotherapies are not currently recommended [3,5]. In patients who have an insufficient response to cDMARDs, biologics are not cost-effective with the 35,000 €/QALY threshold, and with the higher thresholds of 50,000–100,000 €/QALY the evidence of their cost-effective is conflicting. It should be noted that ADA, ETN and IFX, which have been for the longest time on the market, have been assessed in several studies and are consequently associated with a wide range of different ICERs. Meanwhile the narrower ranges of ICER values for ABT and TOC probably reflect the lower number of studies rather than more consistent performance of these agents. Health technology assessment reports provided by independent organisations such as NICE tend to provide higher ICERs than CUAs funded by pharmaceutical companies, due to different premises of the studies. Such publicly funded and in this respect independent reports are not yet available for the newer agents such as TOC, which also may at least in part explain more favourable ICERs. Among the patients with an inadequate response to one TNFi, RTX appears cost-effective with the threshold of 35,000 €/QALY. With the higher thresholds also other TNFis and ABT might be cost effective. These findings are consistent with previous systematic reviews on the current topic [8–10].

We performed this review following current recommendations for systematic literature review of economic evaluations [11]. Standardized methodology is a certain guarantee for the quality and reliability of the current work. Source studies were restricted to CUAs, instead of all CEAs, because QALY as a single measure of the effectiveness enables more accurate comparison of the results. A further aim was to enhance the comparability of the studies by classifying them by previous treatments and comparator treatments. Such a classification seems almost to be necessary because the patient history is a key factor while assessing the external validity and trying to generalize the results and because the comparator treatment has a great impact on ICERs.

The importance of adequate comparator has been previously raised by Tsao and colleagues in their systematic review examining the cost-effectiveness of biologics in comparison to cDMARDs [9]. MTX was the most frequent comparator in the studies included in the current systematic review. MTX is the drug of choice in cDMARD naïve patient population [3]. On the other hand, in patients with MTX monotherapy treatment failure this drug does not represent an adequate treatment option. Instead patients should be treated with other cDMARDs or a combination of cDMARDs they have not received before. In the current study ICERs were assessed using comparator treatments and it seems that CUAs applying adequate comparators may provide rather high ICERs. However, in spite of the general acceptance of MTX as an anchor drug in RA, there is a lack of consensus on the optimal cDMARDs sequence, which poses a problem for CUAs.

It should be noticed that in spite of stratification of patients to subgroups, methodological differences make a comparison of different CUAs difficult. Heterogeneity in time horizons, discount rates, and perspectives were observed, all possibly inducing differences between the studies. For example, it is likely that a CUA with a longer time horizon produces more favourable ICERs compared to ones with shorter time horizons [17,36,43]. While biologics are expensive, they might induce future savings through decreased productivity losses and the lesser need for surgery and inpatient care. A discount rate depreciates the future costs and benefits of the treatment consequently reducing their impact on ICER.

Analyses counting only direct costs give an incomplete view of the pros and cons of different treatments, while various methods used to estimate indirect costs remain controversial. In the current study ICERs based only on direct costs and ICERs based on the inclusion of both direct and indirect costs are provided if they were reported in the original source publication. It is likely that biologics decrease productivity costs because they improve the health status of the patients [5,6]. However, the age and employment status of treated population and the overall labour costs have a major impact on indirect costs, introducing heterogeneity in the ICERs. For example, in China where labour costs are low, Wu *et al*. reported only small differences between ICERs including direct or both direct and indirect costs, while in Sweden much larger differences were observed [25,29,36,38,52]. The method used for the evaluation of productivity costs generate further variation in ICERs when also indirect costs are considered: Van den Hout *et al*. reported ICERs of 147,000€/QALY and 25,000€/QALY for early IFX treatment using friction cost and human capital methods, respectively [19]. For these reasons it is more transparent not to use ICERs with indirect costs when results of different studies are to be compared. Accordingly, conclusions in the current review are based on ICERs including only direct costs. Health service and other costs are always also related to national economy, health policy and price level and thus ICERs cannot directly be generalized when analysing results from different countries.

Different methodologies used for the QALY measures have effect on ICERs. In most studies, the utility scores of the multiattribute utility (MAU) instruments (e.g. EQ-5D) were derived from the Health assessment questionnaire (HAQ) or some other disease specific measures. This is necessary due to the fact that the MAU instruments have been applied in few RCTs, while disease specific measures such as HAQ have been commonly used in RCTs. Application of different formulas for conversions introduce a further source of heterogeneity in ICERs estimates [44]. Different MAU instruments without any conversions produce different utility scores and hence, different ICERs [19]. Standardization of MAU instruments and a validated standard conversion method for missing utility measures would enable better comparison between different CUAs.

In most studies the effectiveness estimates were based on one or several RCTs, representing rather estimates for efficacy. While RCTs are the key source for the efficacy evidence in medicines, they have some weaknesses if applied as source of effectiveness estimates in economic evaluations. Firstly, the results of RCTs are generally better than in the clinical practice because patients are carefully selected and adherence is usually better to RCTs than to regular clinical practices. Consequently, ICERs based on efficacy estimates from RCTs tend to be much lower than those based on observational data as shown by Farahani *et al*. [37]. Secondly, an objective of RCTs is usually to explore an efficacy of a single treatment in comparisons to placebo (or MTX in case of several RCTs studying biologics for RA), rather than compare complex treatment strategies. In contrast, CUAs aim to compare active treatments reflecting real life practices, and therefore indirect comparisons of RCTs are often necessary. However, some CUAs which used effectiveness estimates obtained from several RCTs reported indirect comparisons inadequately. This, restricted clinical evidence and therefore somewhat inconsistent results from CUAs explain that the ranking of biologics remains unclear among patients having inadequate response for cDMARDs [6]. To advance CUAs even further, indirect comparisons could in the future be performed and reported according to current guidelines [56].

The quality of economic evaluations was assessed using two different checklists, and was found to be suboptimal. The quality scores according the BMJ checklist were rather high while Philips’ checklist provided less favorable estimates of the study qualities. The reason for this discrepancy is probably the extensiveness of the Philips’ checklist, which covers several topics not considered in the BMJ checklist. An interesting finding was that quality scores of the studies were not associated with the magnitude of ICER. This is perhaps based on the nature of checklists: a single and simple modeling assumption may have a great impact on ICERs even if its effect on quality scores remains minor. In addition to the quality assessment of the individual studies, we assessed the bias across the CUAs. Only a few of the older conference abstracts identified through the literature search have been published later as a full article, indicating a reporting bias. However, conference abstracts were not included in the current systematic literature review due to incomplete information and problems with quality assessment that may bias their results. The risk of a language bias seems minor based on the small number of non-English papers excluded.

## Conclusions

With the WTP threshold of 35,000 €/QALY, biologics do not seem to be cost-effective among cDMARD naïve patients or cDMARD resistant patients. Among patients with an inadequate response to TNFi(s), RTX seems to be cost-effective. With thresholds of 50,000–100,000 €/QALY biologics might be cost-effective among cDMARD resistant patients.

## Supporting Information

S1 Checklist PRISMA Checklist. (DOCX)

S1 File Search strategy for PubMed. (DOCX)

S2 File Studies excluded after full-text assessment. Duplicate references (n = 7) are excluded from a list.
(DOCX)

S1 Table Inclusion and exclusion criteria. (DOCX)

## References

[1] Alamanos Y, Voulgari PV, Drosos AA. Incidence and prevalence of rheumatoid arthritis, based on the 1987 American College of Rheumatology criteria: a systematic review. Semin Arthritis Rheum. 2006;36: 182–188. 17045630 10.1016/j.semarthrit.2006.08.006

[2] Lundkvist J, Kastäng F, Kobelt G. The burden of rheumatoid arthritis and access to treatment: health burden and costs. Eur J Health Econ. 2008;8: S49–60. 18157732 10.1007/s10198-007-0088-8

[3] Smolen JS, Landewé R, Breedveld FC, Buch M, Burmester G, Dougados M, et al EULAR recommendations for the management of rheumatoid arthritis with synthetic and biological disease-modifying antirheumatic drugs: 2013 update. Ann Rheum Dis. 2014;73: 492–509. 10.1136/annrheumdis-2013-204573 24161836 PMC3933074

[4] Aaltonen KJ, Virkki LM, Malmivaara A, Konttinen YT, Nordström DC, Blom M. Systematic review and meta-analysis of the efficacy and safety of existing TNF blocking agents in treatment of rheumatoid arthritis. PLoS One. 2012;7: e30275 10.1371/journal.pone.0030275 22272322 PMC3260264

[5] Nam JL, Winthrop KL, van Vollenhoven RF, Pavelka K, Valesini G, Hensor EM a, et al Current evidence for the management of rheumatoid arthritis with biological disease-modifying antirheumatic drugs: a systematic literature review informing the EULAR recommendations for the management of RA. Ann Rheum Dis. 2010;69: 976–986. 10.1136/ard.2009.126573 20447957

[6] Nam JL, Ramiro S, Gaujoux-Viala C, Takase K, Leon-Garcia M, Emery P, et al Efficacy of biological disease-modifying antirheumatic drugs: a systematic literature review informing the 2013 update of the EULAR recommendations for the management of rheumatoid arthritis. Ann Rheum Dis. 2014;73: 516–528. 10.1136/annrheumdis-2013-204577 24399231

[7] Drummond M, Sculpher M, Torrance G, Bernie O, Stoddart G. Methods for Economic Evaluation of Health Care Programmes. 3rd ed. New York: Oxford University Press; 2005

[8] Schoels M, Wong J, Scott DL, Zink A, Richards P, Landewé R, et al Economic aspects of treatment options in rheumatoid arthritis: A systematic literature review informing the EULAR recommendations for the management of rheumatoid arthritis. Ann Rheum Dis. 2010;69: 995–1003. 10.1136/ard.2009.126714 20447950

[9] Tsao NW, Bansback NJ, Shojania K, Marra C a. The issue of comparators in economic evaluations of biologic response modifiers in rheumatoid arthritis. Best Pract Res Clin Rheumatol. 2012;26: 659–676. 10.1016/j.berh.2012.07.012 23218430

[10] Van der Velde G, Pham B, Machado M, Ieraci L, Witteman W, Bombardier C, et al Cost-effectiveness of biologic response modifiers compared to disease-modifying antirheumatic drugs for rheumatoid arthritis: a systematic review. Arthritis Care Res(Hoboken). 2011;63: 65–78. 10.1002/acr.20338 20740606

[11] Shemilt I, Mugford M, Byford S, Drummond M, Eisenstein E, Knapp M, et al Incorporating economics evidence In: Higgins JP, Green S, editors. Cochrane Handbook for Systematic Reviews of Interventions. Cochrane Collaboration; 2008 pp. 449–479. 10.1002/14651858.CD004741.pub2

[12] Drummond M, Jefferson T. Guidelines for authors and peer reviewers of economic submissions to the BMJ. BMJ. 1996;313: 275–283. 8704542 10.1136/bmj.313.7052.275 PMC2351717

[13] Philips Z, Ginnelly L, Sculpher M, Claxton K. Review of guidelines for good practice in decision-analytic modelling in health technology assessment. Health Technol Assess. 2004;8: 1–172. 15361314 10.3310/hta8360

[14] Spalding JR, Hay J. Cost effectiveness of tumour necrosis factor-α inhibitors as first-line agents in rheumatoid arthritis. Pharmacoeconomics. 2006;24: 1221–1232. 17129076 10.2165/00019053-200624120-00006

[15] Brennan A, Bansback N, Nixon R, Madan J, Harrison M, Watson K, et al Modelling the cost effectiveness of TNF-α antagonists in the management of rheumatoid arthritis: Results from the British Society for Rheumatology Biologics Registry. Rheumatology(Oxford). 2007;46: 1345–1354. 17562686 10.1093/rheumatology/kem115

[16] Kobelt G, Eberhardt K, Geborek P. TNF inhibitors in the treatment of rheumatoid arthritis in clinical practice: Costs and outcomes in a follow up study of patients with Ra treated with etanercept or infliximab in southern Sweden. Ann Rheum Dis. 2004;63: 4–10. 14672883 10.1136/ard.2003.010629 PMC1754715

[17] Vera-Llonch M, Massarotti E, Wolfe F, Shadick N, Westhovens R, Sofrygin O, et al Cost-effectiveness of abatacept in patients with moderately to severely active rheumatoid arthritis and inadequate response to methotrexate. Rheumatology (Oxford). 2008;47: 535–541. 10.1093/rheumatology/ken007 18356179

[18] Merkesdal S, Kirchhoff T, Wolka D, Ladinek G, Kielhorn A, Rubbert-Roth A. Cost-effectiveness analysis of rituximab treatment in patients in Germany with rheumatoid arthritis after etanercept-failure. Eur J Health Econ. 2010;11: 95–104. 10.1007/s10198-009-0205-y 19967426

[19] Van Den Hout WB, Goekoop-Ruiterman YPM, Allaart CF, Vries-Bouwstra JKD, Hazes JMM, Kerstens PJSM, et al Cost-utility analysis of treatment strategies in patients with recent-onset rheumatoid arthritis. Arthritis Care Res(Hoboken). 2009;61: 291–299. 10.1002/art.24169 19248130

[20] Soini EJ, Puolakka K, Vihervaara V, Kauppi MJ. Cost-effectiveness of adalimumab, etanercept, and tocilizumab as first-line treatments for moderate-to-severe rheumatoid arthritis. J Med Econ. 2012;15: 340–51. 10.3111/13696998.2011.649327 22168785

[21] Davies A, Cifaldi MA, Segurado OG, Weisman MH. Cost-effectiveness of sequential therapy with tumor necrosis factor antagonists in early rheumatoid arthritis. J Rheumatol. 2009;36: 16–25. 10.3899/jrheum.080257 19012363

[22] Clark W, Jobanputra P, Barton P, Burls A. The clinical and cost-effectiveness of anakinra for the treatment of rheumatoid arthritis in adults: a systematic review and economic analysis. Health Technol Assess. 2004;8: 1–117. 15130461 10.3310/hta8180

[23] Welsing PMJ, Severens JL, Hartman M, van Riel PLCM, Laan RFJM. Modeling the 5-year cost effectiveness of treatment strategies including tumor necrosis factor-blocking agents and leflunomide for treating rheumatoid arthritis in the Netherlands. Arthritis Care Res(Hoboken). 2004;51: 964–973. 10.1002/art.20843 15593319

[24] Nguyen CM, Bounthavong M, Mendes MAS, Christopher MLD, Tran JN, Kazerooni R, et al Cost Utility of Tumour Necrosis Factor- a Inhibitors for Rheumatoid Arthritis. Pharmacoeconomics. 2012;30: 575–593. 10.2165/11594990-000000000-00000 22640174

[25] Lekander I, Borgström F, Lysholm J, van Vollenhoven RF, Lindblad S, Geborek P, et al The cost-effectiveness of TNF-inhibitors for the treatment of rheumatoid arthritis in Swedish clinical practice. Eur J Health Econ. 2013;14: 863–873. 10.1007/s10198-012-0431-6 22990378

[26] Lekander I, Borgstrm F, Svarvar P, Ljung T, Carli C, Van Vollenhoven RF. Cost-effectiveness of real-world infliximab use in patients with rheumatoid arthritis in Sweden. Int J Technol Assess Health Care. 2010;26: 54–61. 10.1017/S0266462309990596 20059781

[27] Finckh A, Bansback N, Marra CA, Anis AH, Michaud K, Lubin S, et al Treatment of Very Early Rheumatoid Arthritis With Symptomatic Therapy, Disease-Modifying Antirheumatic Drugs, or Biologic Agents: A Cost-Effectiveness Analysis. Ann Intern Med. 2009;151: 612–621. 10.7326/0003-4819-151-9-200911030-00006 19884622

[28] Chen Y-F, Jobanputra P, Barton P, Jowett S, Bryan S, Clark W, et al A systematic review of the effectiveness of adalimumab, etanercept and infliximab for the treatment of rheumatoid arthritis in adults and an economic evaluation of their cost-effectiveness. Health Technol Assess. 2006;10: 1–248. 17049139 10.3310/hta10420

[29] Wu B, Wilson A, Wang F-f., Wang S-l., Wallace DJ, Weisman MH, et al Cost Effectiveness of Different Treatment Strategies in the Treatment of Patients with Moderate to Severe Rheumatoid Arthritis in China. PLoS One. 2012;7: e47373 10.1371/journal.pone.0047373 23056637 PMC3467255

[30] Schipper LG, Kievit W, den Broeder AA, van der Laar MA, Adang EMM, Fransen J, et al Treatment strategies aiming at remission in early rheumatoid arthritis patients: Starting with methotrexate monotherapy is cost-effective. Rheumatology (Oxford). 2011;50: 1320–1330. 10.1093/rheumatology/ker084 21371999

[31] Kielhorn A, Porter D, Diamantopoulos A, Lewis G. UK cost-utility analysis of rituximab in patients with rheumatoid arthritis that failed to respond adequately to a biologic disease-modifying antirheumatic drug. Curr Med Res Opin. 2008;24: 2639–50. 10.1185/03007990802321683 18687164

[32] Jobanputra P, Barton P, Bryan S, Burls A. The effectiveness of infliximab and etanercept for the treatment of rheumatoid arthritis: A systematic review and economic evaluation. Health Technol Assess. 2002;6: 1–110. 10.3310/hta6210 12387732

[33] Diamantopoulos A, Benucci M, Capri S, Berger W, Wintfeld N, Giuliani G, et al Economic evaluation of tocilizumab combination in the treatment of moderate-to-severe rheumatoid arthritis in Italy. J Med Econ. 2012;15: 576–585. 10.3111/13696998.2012.665110 22313326

[34] Brodszky V, Orlewska E, Péntek M, Kárpáti K, Skoupá J, Gulacsi L. Challenges in economic evaluation of new drugs: Experience with rituximab in Hungary. Med Sci Monit. 2010;16: 1–5. 20037504

[35] Tanno M, Nakamura I, Ito K, Tanaka H, Ohta H, Kobayashi M, et al Modeling and cost-effectiveness analysis of etanercept in adults with rheumatoid arthritis in Japan: A preliminary analysis. Mod Rheumatol. 2006;16: 77–84. 16633926 10.1007/s10165-006-0461-y

[36] Kobelt G, Lindgren P, Singh A, Klareskog L. Cost effectiveness of etanercept(Enbrel) in combination with methotrexate in the treatment of active rheumatoid arthritis based on the TEMPO trial. Ann Rheum Dis. 2005;64: 1174–1179. 15708879 10.1136/ard.2004.032789 PMC1755590

[37] Farahani P, Levine M, Goeree R. A comparison between integrating clinical practice setting and randomized controlled trial setting into economic evaluation models of therapeutics. J Eval Clin Pract. 2006;12: 463–470. 16907691 10.1111/j.1365-2753.2006.00731.x

[38] Kobelt G, Lekander I, Lang A, Raffeiner B, Botsios C, Geborek P. Cost-effectiveness of etanercept treatment in early active rheumatoid arthritis followed by dose adjustment. Int J Technol Assess Health Care. 2011;27: 193–200. 10.1017/S0266462311000195 21736857

[39] Wong JB, Singh G, Kavanaugh A. Estimating the cost-effectiveness of 54 weeks of infliximab for rheumatoid arthritis. Am J Med. 2002;113: 400–408. 12401535 10.1016/s0002-9343(02)01243-3

[40] Wailoo AJ, Bansback N, Brennan A, Michaud K, Nixon RM, Wolfe F. Biologic drugs for rheumatoid arthritis in the Medicare program: a cost-effectiveness analysis. Arthritis Rheum. 2008;58: 939–46. 10.1002/art.23374 18383356

[41] Bansback NJ, Brennan A, Ghatnekar O. Cost effectiveness of adalimumab in the treatment of patients with moderate to severe rheumatoid arthritis in Sweden. Ann Rheum Dis. 2005;64: 995–1002. 15550533 10.1136/ard.2004.027565 PMC1755554

[42] Yuan Y, Trivedi D, Maclean R, Rosenblatt L. Indirect cost-effectiveness analyses of abatacept and rituximab in patients with moderate-to-severe rheumatoid arthritis in the United States. J Med Econ. 2010;13: 33–41. 10.3111/13696990903508021 20001596

[43] Vera-Llonch M, Massarotti E, Wolfe F, Westhovens R, Sofrygin O, Maclean R, et al Cost-Effectiveness of Abatacept in Patients with Moderately to Severely Active Rheumatoid Arthritis and Inadequate Response to Tumor Necrosis Factor- α Antagonists. J Rheumatol. 2008;35: 1745–1753. 18634164

[44] Marra CA, Marion SA, Guh DP, Najafzadeh M, Wolfe F, Esdaile JM, et al Not all “quality-adjusted life years” are equal. J Clin Epidemiol. 2007;60: 616–624. 17493521 10.1016/j.jclinepi.2006.09.006

[45] Lindgren P, Geborek P, Kobelt G. Modeling the cost-effectiveness of treatment of rheumatoid arthritis with rituximab using registry data from Southern Sweden. Int J Technol Assess Health Care. 2009;25: 181–189. 10.1017/S0266462309090230 19331709

[46] Coyle D, Judd M, Blumenauer B, Cranney A, Tugwell P, Well GA. Infliximab and Etanercept in Patients with Rheumatoid Arthritis: A Systematic Review and Economic Evaluation [Technology report no 64]. Ottawa: Canadian Coordinating Office for Health Technology Assessment. 2006. Available: http://www.cadth.ca/en/products/health-technology-assessment/publication/610. Accessed 4 February 2015.

[47] Chiou C-F, Choi J, Reyes CM. Cost-effectiveness analysis of biological treatments for rheumatoid arthritis. Expert Rev Pharmacoecon Outcomes Res. 2004;4: 307–315. 10.1586/14737167.4.3.307 19807313

[48] Canadian Agency for Drugs and Technologies in Health. Clinical and Economic Overview: Biological Response Modifier Agents for Adults with Rheumatoid Arthritis. CADTH Therapeutic Review. 2010. Available: http://www.cadth.ca/media/pdf/TR_RA_Clinical_and_Economic_Overview_e.pdf. Accessed 4 February 2015.

[49] Brennan A, Bansback N, Reynolds A, Conway P. Modelling the cost-effectiveness of etanercept in adults with rheumatoid arthritis in the UK. Rheumatology(Oxford). 2004;43: 62–72. 12890861 10.1093/rheumatology/keg451

[50] Barton P, Jobanputra P, Wilson J, Bryan S, Burls A. The use of modelling to evaluate new drugs for patients with a chronic condition: the case of antibodies against tumour necrosis factor in rheumatoid arthritis. Health Technol Assess. 2004;8: 1–104. 14982655 10.3310/hta8110

[51] Barbieri M, Wong JB, Drummond M. The cost effectiveness of infliximab for severe treatment-resistant rheumatoid arthritis in the UK. Pharmacoeconomics. 2005;23: 607–618. 15960556 10.2165/00019053-200523060-00007

[52] Kobelt G, Jönsson L, Young A, Eberhardt K. The cost-effectiveness of infliximab (Remicade) in the treatment of rheumatoid arthritis in Sweden and the United Kingdom based on the ATTRACT study. Rheumatology(Oxford). 2003;42: 326–335. 12595631 10.1093/rheumatology/keg107

[53] Malottki K, Barton P, Tsourapas A, Uthman AO, Liu Z, Routh K, et al Adalimumab, etanercept, infiximab, rituximab and abatacept for the treatment of rheumatoid arthritis after the failure of a tumour necrosis factor inhibitor: A systematic review and economic evaluation. Health Technol Assess. 2011;15: 1–278. 10.3310/hta15140 PMC4781257 21439251

[54] Hallinen TA, Soini EJ, Eklund K, Puolakka K. Cost-utility of different treatment strategies after the failure of tumour necrosis factor inhibitor in rheumatoid arthritis in the Finnish setting. Rheumatology(Oxford). 2010;49: 767–777. 10.1093/rheumatology/kep425 20100793 PMC2838414

[55] National Institution for Health and Clinical Excellence. Guide to the methods of technology appraisal 2013. 2013. Available: http://publications.nice.org.uk/pmg9. Accessed 4 February 2015.

[56] Hoaglin DC, Hawkins N, Jansen JP, Scott D a, Itzler R, Cappelleri JC, et al Conducting indirect-treatment-comparison and network-meta-analysis studies: report of the ISPOR Task Force on Indirect Treatment Comparisons Good Research Practices: part 2. Value Health. 2011;14: 429–437. 10.1016/j.jval.2011.01.011 21669367

