# Supplementary material for: The Cost-Effectiveness of Biologics for the Treatment of Rheumatoid Arthritis: A Systematic Review
Source: PLoS One. 2015 Mar 17;10(3):e0119683. doi: 10.1371/journal.pone.0119683 (PMC4363598; doi:10.1371/journal.pone.0119683)
Supplement: S1 Checklist — (DOCX) [file pone.0119683.s001.docx]

| **Section/topic** | | **#** | | **Checklist item** | **Reported on page #** |
| --- | --- | --- | --- | --- | --- |
| **TITLE** | | | | |  |
| Title | 1 | | | “The Cost-effectiveness of Biologics for the Treatment of Rheumatoid Arthritis: a Systematic Review” | Title |
| **ABSTRACT** | | | | |  |
| Structured summary | 2 | | “Background & Objectives: Economic evaluations provide information to aid the optimal utilization of limited healthcare resources. Costs of biologics for Rheumatoid arthritis (RA) are remarkably high, which makes these agents an important target for economic evaluations. This systematic review aims to identify existing studies examining the cost-effectiveness of biologics for RA, assess their quality and report their results systematically.  Methods: A literature search covering Medline, Scopus, Cochrane library, ACP Journal club and Web of Science was performed in March 2013. The cost-utility analyses (CUAs) of one or more available biological drugs for the treatment of RA in adults were included. Two independent investigators systematically collected information and assessed the quality of the studies. To enable the comparison of the results, all costs were converted to 2013 euro.  Results: Of the 4890 references found in the literature search, 41 CUAs were included in the current systematic review. While considering only direct costs, the incremental cost-effectiveness ratio (ICER) of the tumor necrosis factor inhibitors (TNFi) ranged from 39,000 to 1 273,000 €/quality adjusted life year (QALY) gained in comparison to conventional disease-modifying antirheumatic drugs (cDMARDs) in cDMARD naïve patients. Among patients with an insufficient response to cDMARDs, biologics were associated with ICERs ranging from 12,000 to 708,000 €/QALY. Rituximab was found to be the most cost-effective alternative compared to other biologics among the patients with an insufficient response to TNFi.  Conclusions: When 35,000 €/QALY is considered as a threshold for the ICER, TNFis do not seem to be cost-effective among cDMARD naïve patients and patients with an insufficient response to cDMARDs. With thresholds of 50,000 to 100,000 €/QALY biologics might be cost-effective among patients with an inadequate response to cDMARDs. Standardization of multiattribute utility instruments and a validated standard conversion method for missing utility measures would enable better comparison between CUAs.” | | Abstract |
| **INTRODUCTION** | | | | |  |
| Rationale | 3 | | “Economic evaluations provide information on the benefits and costs of these expensive treatments to aid the optimal utilization of limited healthcare resources [7]. Cost-effectiveness analysis (CEA) is the most typical form of economic evaluation for health care interventions. In CEA, costs and effectiveness of two or more treatments are compared. The costs are measured in monetary units and effectiveness in natural units, for example in life years or pain free days. Cost-utility analysis (CUA) is a subtype of CEA, applying quality adjusted life years (QALY) as a measure of effectiveness. The primary outcome measure in CUAs is incremental cost-effectiveness ratio ICER, which describes the ratio of the additional costs of a treatment (compared to an alternative) to QALYs gained. An ICER is not reported if one treatment is both cheaper and more effective than another, e.g. if it is dominant.  Biologics for RA are an important target for economic evaluations because of the associated high costs. Previous systematic reviews suggest that biologics might be cost-effective at the willingness to pay (WTP) threshold of 50,000 - 100,000 $/QALY among patients with insufficient treatment response to cDMARD but not in cDMARD naïve patients [8–10]. However, these reviews involve some weaknesses such as lack of quality assessment [9], insufficient reporting of study characteristics [8] or omission of between-biologics comparison [10].” | | Introduction |
| Objectives | 4 | | “The aim of our systematic review is to identify all existing studies examining the cost-utility of one or more biologics for RA in adults, assess their quality and report their results systematically” | | Introduction |
| **METHODS** | | | | |  |
| Protocol and registration | 5 | | - | | Not applicable |
| Eligibility criteria | 6 | | “Of the remaining references, the CUAs of one or more currently available biologics for the treatment of RA in adults were selected using a pre-defined inclusion and exclusion criteria (S3 Table). The evaluation for inclusion was conducted independently by two persons (JJ and KA) at first by titles and afterwards by full-text. In case of disagreement, a third opinion (MB) was requested. Studies without active comparison treatment (cDMARDs or other biologics) or QALYs as measure of effectiveness were excluded from this systematic review. Reporting of ICER was required, if applicable. Studies published only as conference abstracts and articles without English full-text were excluded.” | | Methods (Study selection), S3 Table |
| Information sources | 7 | | “The search covering Medline, SCOPUS (including EMBase), Cochrane library (Database of Abstracts of Reviews of Effects, Health Technology Assessment Database, Cochrane Database of Systematic Reviews, NHS Economic Evaluation Database, Cochrane Central Register of Controlled Trials and Cochrane Methodology Register), ACP Journal club and Web of science was executed in March 2013 using a search strategy developed with a librarian.” | | Methods (Literature search) |
| Search | 8 | | “The complete search strategy for PubMed is presented in S2 File.” | | Methods (Literature search), File S2 |
| Study selection | 9 | | “All references identified by the literature search were imported to reference management software (Refworks), where duplicate records were removed. Of the remaining references, the CUAs of one or more currently available biologics for the treatment of RA in adults were selected using a pre-defined inclusion and exclusion criteria (S3 Table). The evaluation for inclusion was conducted independently by two persons (JJ and KA) at first by titles and afterwards by full-text. In case of disagreement, a third opinion (MB) was requested.” | | Methods (Study selection) |
| Data collection process | 10 | | “Two assessors (JJ and SH) independently extracted the data and discrepancies were resolved by consulting the third investigator (MB).” | | Methods (Data collection) |

| **Section/topic** | **#** | **Checklist item** | **Reported on page #** |
| --- | --- | --- | --- |
| Data items | 11 | “The Data on patients, interventions, controls, study design (country, perspective, time horizon, the year of resource utilization, included costs, discount rate, the source of effectiveness, the instrument for utility measures, study funding) and outcomes were extracted using a Microsoft Excel - based collection form. “ | Methods (Data collection) |
| Risk of bias in individual studies | 12 | "As currently recommended, the quality of economic evaluations included was assessed using the British Medical Journal (BMJ) checklist and in addition, the Philips` checklist for modelling studies [11–13]. Two investigators (JJ and SH) assessed the quality of the studies independently and the third investigator (MB) was consulted when necessary. BMJ checklist involves 35 items and Philips’ checklist 57 items. Quality scores based on fulfilment of items and average percentages of the applicable criteria met were calculated. To assess the relative quality of the studies we divided studies in three categories (good, adequate and poor quality) ranking them by using the average percentages.” | Methods (Quality assessment) |
| Summary measures | 13 | “To enable a comparison of the results, all of the reported costs were converted to euro using the European Central Bank exchange rates (http://sdw.ecb.europa.eu) and adjusted to the price level of the year 2013 using the price index of Health care expenditure in Finland (Statistics Finland). ICERs including only direct costs were considered primary results due to differences in the ways indirect costs (e.g. productivity losses) were calculated in studies. In addition, ICERs including both direct and indirect costs were presented as secondary outcomes, if reported in the original studies.” | Methods (Representation of results) |
| Synthesis of results | 14 | “The quantitative synthesis of the results of the studies included is not possible owing to heterogeneous study designs. Results of the CUAs included were stratified into five subgroups by type of drug used, previous treatments and response to them, and the comparator treatment as follows: 1) Biologics for cDMARDs naive patients, 2) Biologics compared with cDMARD in patients with an inadequate response to one or several cDMARDs, 3) Biologics compared with other biologics among patients with an inadequate response to cDMARDs, 4) Biologics compared with cDMARDs among patients with an inadequate response to TNFi(s) and 5) Biologics compared with other biologics among patients with an inadequate response to TNFi(s). Further, CUAs were stratified according to adequateness of the comparator treatment. Adequate comparator was defined as a cDMARD not used before [3].” | Methods (Representation of results) |
| Risk of bias across studies | 15 | - | - |
| Additional analyses | 16 | - | Not applicable |
| **RESULTS** | | |  |
| Study selection | 17 | “Altogether, 4653 non-duplicate references were identified with the literature search, of which 3113 were excluded during title and abstract screening (Fig. 1). After the assessment of 237 full-text articles, 41 were included in the current review.” | Results, Fig 1. |
| Study characteristics | 18 | “The 41 CUAs included were published 2002-2013 [14–54]. One study was based on empiric cost and effectiveness data from a randomised controlled trial (RCT) [19], two on observational data [16,37] while the remaining 38 studies used a modelling approach with multiple data sources [14,15,17,18,20–36,38–54]. In 33 of the 38 modelling studies effectiveness estimates were derived from one or more RCTs, while five modelling studies applied effectiveness obtained from national registers. A summary of the characteristics of the CUAs included is shown in Table 1.” | Results (Characteristics of studies included in the current review), Table 1 |
| Risk of bias within studies | 19 | “The average quality scores of the 41 studies included in the present review were 25.7 out of 35 (range 17 to 31) and 32.3 out of 57 (range 16 to 46) when evaluated using BMJ checklist and Philips’ list, respectively (Table 7).” | Results (Quality of the included studies), Table 7 |
| Results of individual studies | 20 | Tables 2-6 | Results, Tables 2-6 |
| Synthesis of results | 21 | - | Not applicable |
| Risk of bias across studies | 22 | - | - |
| Additional analysis | 23 | - | Not applicable |
| **DISCUSSION** | | |  |
| Summary of evidence | 24 | “We performed a systematic literature review of cost-effectiveness of biologics used for the treatment of RA. After the literature search and the selection process of the initially identified reports, 41 original articles were included in the current review. While considering only direct costs, the ICERs of the TNFis ranged from 39,000 to 1 273,000 €/ QALY in comparison to cDMARD in patients naïve to cDMARDs. Among patients with an inadequate response to cDMARDs, biologics were associated with ICERs ranging from 12,000 to 708,000 €/QALY. In this setting, none of the biologics appeared to be more cost-effective than any of the others. ICERs for the second line biologics ranged from 26,000 to 1 347,000 €/QALY in comparison to cDMARDs among patients with an inadequate response to TNFi. In this patient subgroup RTX was the most and ANA the least cost-effective biologic. The quality assessment revealed several problems, namely insufficient reporting of data sources and problematic methodological details, which possibly reduce the validity of the results.” | Discussion |
| Limitations | 25 | “It should be noted that ADA, ETN and IFX, which have been for the longest time on the market, have been assessed in several studies and are consequently associated with a wide range of different ICERs. Meanwhile the narrower ranges of ICER values for ABT and TOC probably reflect the lower number of studies rather than more consistent performance of these agents. Health technology assessment reports provided by independent organisations such as NICE tend to provide higher ICERs than CUAs funded by pharmaceutical companies, due to different premises of the studies. Such publicly funded and in this respect independent reports are not yet available for the newer agents such as TOC, which also may at least in part explain more favourable ICERs.”  “The importance of adequate comparator has been previously raised by Tsao and colleagues in their systematic review examining the cost-effectiveness of biologics in comparison to cDMARDs [9]. MTX was the most frequent comparator in the studies included in the current systematic review. MTX is the drug of choice in cDMARD naïve patient population [3]. On the other hand, in patients with MTX monotherapy treatment failure this drug does not represent an adequate treatment option. Instead patients should be treated with other cDMARDs or a combination of cDMARDs they have not received before. In the current study ICERs were assessed using comparator treatments and it seems that CUAs applying adequate comparators may provide rather high ICERs. However, in spite of the general acceptance of MTX as an anchor drug in RA, there is a lack of consensus on the optimal cDMARDs sequence, which poses a problem for CUAs.”  “It should be noticed that in spite of stratification of patients to subgroups, methodological differences make a comparison of different CUAs difficult. Heterogeneity in time horizons, discount rates, and perspectives were observed, all possibly inducing differences between the studies. For example, it is likely that a CUA with a longer time horizon produces more favourable ICERs compared to ones with shorter time horizons [17,36,43]. While biologics are expensive, they might induce future savings through decreased productivity losses and the lesser need for surgery and inpatient care. A discount rate depreciates the future costs and benefits of the treatment consequently reducing their impact on ICER.  Analyses counting only direct costs give an incomplete view of the pros and cons of different treatments, while various methods used to estimate indirect costs remain controversial. In the current study ICERs based only on direct costs and ICERs based on the inclusion of both direct and indirect costs are provided if they were reported in the original source publication. It is likely that biologics decrease productivity costs because they improve the health status of the patients [5,6]. However, the age and employment status of treated population and the overall labour costs have a major impact on indirect costs, introducing heterogeneity in the ICERs. For example, in China where labour costs are low, Wu et al. reported only small differences between ICERs including direct or both direct and indirect costs, while in Sweden much larger differences were observed [25,29,36,38,52]. The method used for the evaluation of productivity costs generate further variation in ICERs when also indirect costs are considered: Van den Hout et al. reported ICERs of 147,000€/QALY and 25,000€/QALY for early IFX treatment using friction cost and human capital methods, respectively [19]. For these reasons it is more transparent not to use ICERs with indirect costs when results of different studies are to be compared. Accordingly, conclusions in the current review are based on ICERs including only direct costs. Health service and other costs are always also related to national economy, health policy and price level and thus ICERs cannot directly be generalized when analysing results from different countries.  Different methodologies used for the QALY measures have effect on ICERs. In most studies, the utility scores of the multiattribute utility (MAU) instruments (e.g. EQ-5D) were derived from the Health assessment questionnaire (HAQ) or some other disease specific measures. This is necessary due to the fact that the MAU instruments have been applied in few RCTs, while disease specific measures such as HAQ have been commonly used in RCTs. Application of different formulas for conversions introduce a further source of heterogeneity in ICERs estimates [44]. Different MAU instruments without any conversions produce different utility scores and hence, different ICERs [19]. Standardization of MAU instruments and a validated standard conversion method for missing utility measures would enable better comparison between different CUAs.  In most studies the effectiveness estimates were based on one or several RCTs, representing rather estimates for efficacy. While RCTs are the key source for the efficacy evidence in medicines, they have some weaknesses if applied as source of effectiveness estimates in economic evaluations. Firstly, the results of RCTs are generally better than in the clinical practice because patients are carefully selected and adherence is usually better to RCTs than to regular clinical practices. Consequently, ICERs based on efficacy estimates from RCTs tend to be much lower than those based on observational data as shown by Farahani et al. [37]. Secondly, an objective of RCTs is usually to explore an efficacy of a single treatment in comparisons to placebo (or MTX in case of several RCTs studying biologics for RA), rather than compare complex treatment strategies. In contrast, CUAs aim to compare active treatments reflecting real life practices, and therefore indirect comparisons of RCTs are often necessary. However, some CUAs which used effectiveness estimates obtained from several RCTs reported indirect comparisons inadequately. This, restricted clinical evidence and therefore somewhat inconsistent results from CUAs explain that the ranking of biologics remains unclear among patients having inadequate response for cDMARDs [6]. To advance CUAs even further, indirect comparisons could in the future be performed and reported according to current guidelines [56].  The quality of economic evaluations was assessed using two different checklists, and was found to be suboptimal. The quality scores according the BMJ checklist were rather high while Philips’ checklist provided less favorable estimates of the study qualities. The reason for this discrepancy is probably the extensiveness of the Philips’ checklist, which covers several topics not considered in the BMJ checklist. An interesting finding was that quality scores of the studies were not associated with the magnitude of ICER. This is perhaps based on the nature of checklists: a single and simple modeling assumption may have a great impact on ICERs even if its effect on quality scores remains minor. In addition to the quality assessment of the individual studies, we assessed the bias across the CUAs. Only a few of the older conference abstracts identified through the literature search have been published later as a full article, indicating a reporting bias. However, conference abstracts were not included in the current systematic literature review due to incomplete information and problems with quality assessment that may bias their results. The risk of a language bias seems minor based on the small number of non-English papers excluded.“ | Discussion |
| Conclusions | 26 | “With the WTP threshold of 35,000 €/QALY, biologics do not seem to be cost-effective among cDMARD naïve patients or cDMARD resistant patients. Among patients with an inadequate response to TNFi(s), RTX seems to be cost-effective. With thresholds of 50,000-100,000 €/QALY biologics might be cost-effective among cDMARD resistant patients.” | Conclusions |
| **FUNDING** | | |  |
| Funding | 27 | JJ has received 22,000 € grant from Research Foundation of the University of Helsinki  (http://www.helsinki.fi/tiedesaatio/grants/)  The funders had no role in study design, data collection and analysis, decision to publish, or preparation of the manuscript. | Submission |

*From:*  Moher D, Liberati A, Tetzlaff J, Altman DG, The PRISMA Group (2009). Preferred Reporting Items for Systematic Reviews and Meta-Analyses: The PRISMA Statement. PLoS Med 6(6): e1000097. doi:10.1371/journal.pmed1000097

For more information, visit: **www.prisma-statement.org**.
