# Supplementary material for: The Cost-Effectiveness of Biologics for the Treatment of Rheumatoid Arthritis: A Systematic Review
Source: PLoS One. 2015 Mar 17;10(3):e0119683. doi: 10.1371/journal.pone.0119683 (PMC4363598; doi:10.1371/journal.pone.0119683)
Supplement: S1 File — (DOCX) [file pone.0119683.s002.docx]

**S1 File. Search strategy for PubMed.**

(((adalimumab OR humira OR etanercept OR enbrel OR rituximab OR (rituxan) OR mabthera OR infliximab OR remicade OR anakinra OR kineret OR abatacept OR orencia OR tocilizumab OR roactemra OR actemra OR golimumab OR simponi OR certolizumab OR certolizumab pegol OR certolizumab pegol OR cimzia OR TNF OR tumor necrosis factor OR tumor necrosis factor OR tumor necrosis factor alpha OR tnf alpha OR tnfalpha OR anti-tnf OR anti tnf OR anti tumor necrosis factor OR anti tumor necrosis factor alpha OR antitumor necrosis factor OR tnf blocker OR tnf blocker OR tumor necrosis factor blocker OR tnf alpha blocker OR tumor necrosis factor alpha blocker OR biologics OR biological agent OR biologic agent OR interleukin 1 receptor antagonist protein OR interleukin 1 receptor antagonist OR monoclonal antibodies OR biological therapy)) AND (rheumatic diseases OR arthritis, rheumatoid)) AND (((((cost-benefit)) OR (economic) OR (cost-effectiveness) OR (cost effectiveness) OR (cost effectiveness) OR (cost-utility) OR cost utility OR (cost utility) OR (quality-adjusted life years) OR (quality adjusted lifeyears) OR qaly OR (cost benefit analysis) OR (cost benefit analysis) OR (cost effectiveness analysis) OR (cost effectiveness analysis) OR (cost effectiveness analysis))) OR ((quality of life) OR (utility) OR (health related quality of life) OR (hrqol)))
