# Supplementary material for: The Cost-Effectiveness of Biologics for the Treatment of Rheumatoid Arthritis: A Systematic Review
Source: PLoS One. 2015 Mar 17;10(3):e0119683. doi: 10.1371/journal.pone.0119683 (PMC4363598; doi:10.1371/journal.pone.0119683)
Supplement: S2 File — Duplicate references (n = 7) are excluded from a list. (DOCX) [file pone.0119683.s003.docx]

**S4 File. Studies excluded after full-text assessment.** Duplicate references (n=7) are excluded from a list.

1. Aagren M, Davies a, Pang F, Winding B (2006) Par10 the Cost-Utility of Adalimumab (Humira®) in Patients With Rheumatoid Arthritis (Ra) in Denmark. Value Heal 9: A223. Available: http://linkinghub.elsevier.com/retrieve/pii/S109830151063269X. Accessed 15 May 2013.

2. Aagren M, Davies A, Winding B, Cifaldi M, Pang F (2006) The Cost-Effectiveness of Adalimumab (HUMIRA®) in Patients with Rheumatoid Arthritis (RA) in Denmark. Scand J Rheumatol 35: 15–15.

3. Adams RC, Walsh C, Schmitz S, Barton P, Barry M (2011) PMS51 A Cost Utility Analysis of Anti-TNF Agents for the Treatment of Rheumatoid Arthritis. Value Heal 14: A311–A312. Available: http://linkinghub.elsevier.com/retrieve/pii/S1098301511019991. Accessed 15 May 2013.

4. Agarwal S, Sansgiry S, Johnson M (2009) Pms23 an Economic Analysis Comparing Three Combinations of Tumor Necrosis Factor-Alpha Agents for the Treatment of Rheumatoid Arthritis. Value Heal 12: A67–A68. Available: http://linkinghub.elsevier.com/retrieve/pii/S1098301510733994. Accessed 15 May 2013.

5. Aguirre a, García E, Bierschwale H, Prado M (2009) Pms4 Costo-Efectividad Del Uso De Etanercept Vs Los Anticuerpos Monoclonales Anti-Tnf En Pacientes Con Artritis Reumatoide En Costa Rica. Value Heal 12: A522. Available: http://linkinghub.elsevier.com/retrieve/pii/S1098301510754802. Accessed 15 May 2013.

6. Alfonso-Cristancho R, Aiello EC, Roa CN (2011) Pms25 Cost-Effectiveness of Abatacept or Infliximab in Rheumatoid Arthritis in Colombia. Value Heal 14: A127. Available: http://linkinghub.elsevier.com/retrieve/pii/S1098301511008497. Accessed 15 May 2013.

7. Alfonso-Cristancho R, Serra N, Aiello EC, Roa CN (2011) Pms21 Abatacept or Infliximab for Rheumatoid Arthritis in Venezuela? a Cost-Effectiveness Analysis. Value Heal 14: A126–A127. Available: http://linkinghub.elsevier.com/retrieve/pii/S109830151100845X. Accessed 15 May 2013.

8. Allaart CF, Breedveld FC, Dijkmans BAC (2007) Treatment of recent-onset rheumatoid arthritis: Lessons from the BeSt study. J Rheumatol 34: 25–33. Available: http://www.scopus.com/inward/record.url?eid=2-s2.0-35948956315&partnerID=40&md5=d88ffe72ef61e31a1df93368aa593b58.

9. Alves M, Carvalho Jr F, Litalien G (2008) Pms18 Cost-Effectiveness Ofabatacept in Patients With Moderatelyto Severely Active Rheumatoid Arthritis and Inadequate Response To Methotrexate in Brazil. Value Heal 11: A260. Available: http://linkinghub.elsevier.com/retrieve/pii/S1098301510708202. Accessed 15 May 2013.

10. Bagust A, Boland A, Hockenhull J, Fleeman N, Greenhalgh J, et al. (2009) Rituximab for the treatment of rheumatoid arthritis. Health Technol Assess 13: 23–29.

11. Bansback N (2003) Cost-effectiveness of adalimumab (Humira (TM)), Abbott) in patients with rheumatoid arthritis: A Swedish analysis. Arthritis Rheum 48: S611.

12. Bansback N (2003) Cost effectiveness of adalimumab (Humira (TM), Abbott) in the treatment of patients with moderate to severe rheumatoid arthritis (RA). Ann Rheum Dis 48: S215.

13. Bansback N (2002) A threshold cost-effectiveness comparison of infliximab and etanercept in adults with rheumatoid arthritis in the UK. Arthritis Rheum 46: S582.

14. Bansback N (2004) Cost-effectiveness analyses of TNF antagonists in rheumatoid arthritis: A review. Ann Rheum Dis 50: S510.

15. Bansback NJ, Regier DA, Ara R, Brennan A, Shojania K, et al. (2005) An overview of economic evaluations for drugs used in rheumatoid arthritis: Focus on tumour necrosis factor-α antagonists. Drugs 65: 473–496. Available: http://www.scopus.com/inward/record.url?eid=2-s2.0-16344370052&partnerID=40&md5=0c500dbbc6df859398f514215580cd42.

16. Bansback N, Brennan A, Sengupta N, Pang F (2004) The Cost-Effectiveness Of Adalimumab (Humira) In Patients With Rheumatoid Arthritis: A Uk Analysis. J Orthop Traumatol 50 Suppl 2: 77–77. Available: http://www.ncbi.nlm.nih.gov/pubmed/23484739.

17. Bansback N, Brennan a, Sengupta N (2004) Par5 Cost-Effectiveness of Adalimumab (Humiratm) in the Treatment of Us Patients With Rheumatoid Arthritis (Ra). Value Heal 7: 241. Available: http://linkinghub.elsevier.com/retrieve/pii/S1098301510621298. Accessed 15 May 2013.

18. Barbieri M, Drummond MF, Junoy JP, Gómez MAC, García FJB, et al. (2007) Critical appraisal of pharmacoeconomic studies comparing TNF-α antagonists for rheumatoid arthritis treatment. Expert Rev Pharmacoeconomics Outcomes Res 7: 613–626. Available: http://www.scopus.com/inward/record.url?eid=2-s2.0-37649022658&partnerID=40&md5=85c2a2aa806239aed8a3200947a6586a.

19. Becerra Rojas F, Benites Chacaltana C, Aiello EC, Zingoni C, Bergman G, et al. (2011) Pms33 Cost Effectiveness of Abatacept in Comparison With Other Biologic Therapies for the Treatment of Moderate To Severe Active Rheumatoid Arthritis Patients Who Have Failed To Methotrexate Based Treatment At Essalud in 2010. Value Heal 14: A129. Available: http://linkinghub.elsevier.com/retrieve/pii/S1098301511008576. Accessed 15 May 2013.

20. Belevitin AB, Tyrenko V V., Grigor’eva OA, Bologov SG (2010) [Economic assessment of the use of biological medications in military medicine].

21. Benedict a., Kongnakorn T, Shaw JW, Marcellusi A, Vanness DJ, et al. (2012) PMS46 Economic Evaluation of Adalimumab for the Treatment of Early-and Late-Stage Rheumatoid Arthritis in Italy. Value Heal 15: A447. Available: http://linkinghub.elsevier.com/retrieve/pii/S1098301512031142. Accessed 15 May 2013.

22. Benucci M, Li Gobbi F, Sabadinit L, Saviola G, Baiardi P, et al. (2009) Biologicaltherapy In Rheumatoid Arthritis In Clinical Practice: Cost-Effectiveness Analysis Of Sub-Cutaneous Anti-Tnf a Treatments In Italian Patients. Int J Immunopathol Pharmacol 22: 1147–1152.

23. Benucci M, Saviola G, Manfredi M, Sarzi-Puttini P, Atzeni F (2011) Cost effectiveness analysis of disease-modifying antirheumatic drugs in rheumatoid arthritis. A systematic review literature. Int J Rheumatol 2011. Available: http://www.scopus.com/inward/record.url?eid=2-s2.0-84855585686&partnerID=40&md5=7bfe444a3f52cae6274d057107de5665.

24. Benucci M, Saviola G, Baiardi P, Manfredi M (2011) Cost-effectiveness treatment with Rituximab in patients with rheumatoid arthritis in real life. Rheumatol Int 31: 1465–1469. Available: http://www.ncbi.nlm.nih.gov/pubmed/20473760. Accessed 15 May 2013.

25. Beresniak a, Baerwald C, Zeidler H, Gromnica-lhle E, Kruger K, et al. (2009) Pms40 Cost-Effectiveness Simulation Model of Biologic Strategies for the Treatment of Moderate To Severe Rheumatoid Arthritis Based on Disease Activity in Germany. Value Heal 12: A440. Available: http://linkinghub.elsevier.com/retrieve/pii/S1098301510751792. Accessed 15 May 2013.

26. Beresniak a, Hamuryudan V, Inane M, Pay S, Yazici H, et al. (2009) Pms39 Sequential Cost-Effectiveness Modelling of Different Biologic Strategies in Rheumatoid Arthritis in Turkey. Value Heal 12: A440. Available: http://linkinghub.elsevier.com/retrieve/pii/S1098301510751780. Accessed 15 May 2013.

27. Bozonnat M, Roch F, Cohen J, Al. E (2004) Preliminary medico-economic results at week 54 of a cohort of 613 rheumatoid arthritis (RA) patients treated with infliximab. Ann Rheum Dis 63: 510.

28. Brennan A, Bansback NJ, Nixon R, Madan J, Symmons D (2006) The Cost Effectiveness Of Anti-Tnf Antagonists In Rheumatoid Arthritis: Results From The British Society For Rheumatology Biologics Registry. Ann Rheum Dis 65: 179.

29. Brennan A, Bansback N, Conway P, Reynolds A (2002) Modelling the Cost-Effectiveness of Etanercept in Adults with Rheumatoid Arthritis in the Uk– Treatment. Rheumatology 41: 91–91.

30. Brennan A, Bansback N, Conway P, Reynolds A V (2001) Modelling the cost-effectiveness of etanercept in adults with rheumatoid arthritis (RA) in the UK. 44: S157.

31. Brodszky V, Pentek M, Majer I, Karpati K GL (2007) Abatacept in the treatment of rheumatoid arthritis; systematic review and economic evaluation.

32. Brodszky V, Borgström F, Arnetorp S, Péntek M, Gulácsi L (2008) Pms71 a Cost-Effectiveness Assessment of Abatacept for the Treatment of Rheumatoid Arthritis in Hungary. Value Heal 11: A549–A550. Available: http://linkinghub.elsevier.com/retrieve/pii/S109830151066812X. Accessed 15 May 2013.

33. Brodszky V, Péntek M, Karpati K, Boncz I, Sebestyén a, et al. (2007) Par24 Cost-Utility Analysis of Rituximab Treatment in Rheumatoid Arthritis After Anti-Tnf-Alfatherapy in Hungary. Value Heal 10: A250. Available: http://linkinghub.elsevier.com/retrieve/pii/S109830151064939X. Accessed 20 May 2013.

34. Brodszky V, Pentek M, Majer I, Karpati K, Gulacsi L (2006) Rituximab in patients with rheumatoid arthritis: systematic review and economic evaluation.

35. Brodszky V, Pentek M, Majer I, Karpati K, Gulasci L (2007) Economic evaluation of the first TNF‐alpha inhibitor therapy in patients with rheumatoid arthritis.

36. Bynum L, Joshi N (2010) Pms21 Cost—Effectiveness of Rituximab Vs Adalimumab in the Treatment of Moderate To Severe Rheumatoid Arthritis. Value Heal 13: A126. Available: http://linkinghub.elsevier.com/retrieve/pii/S1098301510726097. Accessed 20 May 2013.

37. Carlos F (2010) Pms38 Cost-Effectiveness of Rituximab Versus Alternative Anti-Tumor Necrosis Factor (Tnf) Therapy After Previous Failure of One Anti-Tnf Agent for Treatment of Rheumatoid Arthritis in Mexico. Value Heal 13: A309. Available: http://linkinghub.elsevier.com/retrieve/pii/S109830151172191X. Accessed 20 May 2013.

38. Carlos F (2010) Pms36 Cost-Effectiveness of Tocilizumab for the Management of Patients With Active Rheumatoid Arthritis Despite Previous Dmard Therapy in Costa Rica. Value Heal 13: A309. Available: http://linkinghub.elsevier.com/retrieve/pii/S1098301511721891. Accessed 20 May 2013.

39. Carlos F (2010) Pms39 Cost-Effectiveness of Rituximab Versus Alternative Anti-Tumor Necrosis Factor (Tnf) Therapy After Previous Failure of One Anti-Tnf Agent for Treatment of Rheumatoid Arthritis in Costa Rica. Value Heal 13: A309. Available: http://linkinghub.elsevier.com/retrieve/pii/S1098301511721921. Accessed 20 May 2013.

40. Carlos F, Aguirre a., Peláez-Ballestas I, Ramos E (2011) PMS5 analisis De Costo-Efectividad De Agentes Biologicos En El Tratamiento De Pacientes Con Artritis reumatoide Activa Y respuesta insuficiente A Farme Tradicionales Desde La perspectiva Del Sistema Publico De Salud En México. Value Heal 14: A562. Available: http://linkinghub.elsevier.com/retrieve/pii/S1098301511032384. Accessed 20 May 2013.

41. Carlos F, Clark P (2011) PMS12 Evaluacion Economica De Rituximab versus anti-Tnf En Pacientes Con Artritis reumatoide Y Falla Previa A anti-Tnf En México. Value Heal 14: A563. Available: http://linkinghub.elsevier.com/retrieve/pii/S1098301511032451. Accessed 20 May 2013.

42. Carlos F, Aguirre a, Peláez-Ballestas I (2010) Pms33 Cost-Effectiveness of Tocilizumab for the Management of Patients With Active Rheumatoid Arthritis Despite Previous Dmard Therapy in Mexico. Value Heal 13: A308. Available: http://linkinghub.elsevier.com/retrieve/pii/S1098301511721866. Accessed 20 May 2013.

43. Carlson JJ, Ogale S, Dejonckheere F, Sullivan S (2012) Cost-Effectiveness of Tocilizumab Monotherapy Vs. Adalimumab Monotherapy in the Treatment of Severe Active Rheumatoid Arthritis. Arthritis Rheum 64: 1037–1038.

44. Chen Y-F, Jobanputra P, Barton P, Jowett S, Bryan S, et al. (2006) A systematic review of the effectiveness of adalimumab, etanercept and infliximab for the treatment of rheumatoid arthritis in adults and an economic evaluation of their cost-effectiveness. Health Technol Assess (Rockv) 10: iii–138. Available: http://www.scopus.com/inward/record.url?eid=2-s2.0-33845482745&partnerID=40&md5=52509f9928cd19a923d7bd737ca4c52e.

45. Connock M, Tubeuf S, Malottki K, Uthman A, Round J, et al. (2010) Certolizumab pegol (CIMZIA®) for the treatment of rheumatoid arthritis. Health Technol Assess 14: 1–10.

46. Creemers MC, Kievit W, Flendrie M, Hoogen FH Van den, Jong AJ De, et al. (2005) Cost-Effectiveness Of Two Tnf Inhibitors, Etanercept And Infliximab, In Patients With Rheumatoid Arthritis In Daily Clinical Practice In The Netherlands. Ann Rheum Dis 64: 430.

47. Davies A (2006) Cost effectiveness of five biologics in the treatment of long-standing moderate to severe rheaumatoid arthritis. Ann Rheum Dis 54: S336–S337.

48. Davies AS, Hutton J, Weisman MH, Cifaldi MA, Pang F (2005) Cost Effectiveness Of Adalimumab (Humira®) Plus Methotrexate Vs. Etanercept (Enbrel) Plus Methotrexate In Moderate To Severe Rheumatoid Arthritis (Ra). Ann Rheum Dis 64: 68.

49. Davies A, Cifaldi MA, Weisman MH, Segurado OG (2006) Cost Effectiveness Of The 3 Tnf Antagonists Vs. Abatacept In The Treatment Of Moderate To Severe Rheumatoid Arthritis (Ra). Ann Rheum Dis 65: 278.

50. Davies AS, Hutton J, Cifaldi MA, Pang F, Spencer-Green G (2005) The Cost Utility Of Adalimumab (Humira®) And Infliximab (Remicade) In The Treatment Of Patients With Early Rheumatoid Arthritis (Ra): Us Analysis. Ann Rheum Dis 64: 393.

51. Davies AS, Hutton J, Cifaldi MA, Pang F, Spencer-Green G (2005) The Cost Effectiveness Of Adalimumab (Humira®) Vs. Infliximab (Remicade) In Achieving Clinical Response At 1 And 3 Years In Patients With Early Rheumatoid Arthritis (Ra): Us Analysis. Ann Rheum Dis 64: 393–394.

52. De Vita S, Giuliani G, Diamantopoulos a, Brown B, Kielhorn a (2006) Par7 Modelling of the Cost-Effectiveness of Rituximab for Treatment of Rheumatoid Arthritis in Italy. Value Heal 9: A26. Available: http://linkinghub.elsevier.com/retrieve/pii/S1098301510643884. Accessed 20 May 2013.

53. Desai RJ, Rao J, Biddle a. K (2012) PMS31 Cost-Effectiveness Analysis of Golimumab for the Treatment of Rheumatoid Arthritis. Value Heal 15: A39. Available: http://linkinghub.elsevier.com/retrieve/pii/S1098301512002859. Accessed 20 May 2013.

54. Diamantopoulos a., Andrade S, Bernardo a., Branco J, Inês L, et al. (2011) PMS30 Cost-Effectiveness of Tocilizumab Compared to Standard Therapeutic Sequences for the Treatment of Moderate/Severe Rheumatoid Arthritis (RA) Patients in Portugal. Value Heal 14: A307. Available: http://linkinghub.elsevier.com/retrieve/pii/S1098301511019784. Accessed 20 May 2013.

55. Diamantopoulos a, Wintfeld N, Giuliani G (2009) Pms54 Cost-Effectiveness of Tocilizumab Compared To Standard of Care for the Treatment of Moderate/Severe Rheumatoid Arthritis (Ra) in Italy. Value Heal 12: A443. Available: http://linkinghub.elsevier.com/retrieve/pii/S1098301510751925. Accessed 20 May 2013.

56. Doan Q V, Chiou C-F, Dubois RW (2006) Review of Eight Pharmacoeconomic Studies of the Value of Biologic DMARDs (Adalimumab, Etanercept, and Infliximab) in the Management of Rheumatoid Arthritis. J Manag Care Pharm 12: 555–569.

57. Du Pan SM, Gabay C, Finckh A (2007) A systematic review of infl iximab in the treatment of early rheumatoid arthritis. 3: 905–911.

58. Emery P, Taylor P, Isaacs J, Drost P, Dupont D, et al. (2009) Pms35 Cost-Effectiveness of Biologic Therapeutic Sequences for Rheumatoid Arthritis in the Uk. Value Heal 12: A439. Available: http://linkinghub.elsevier.com/retrieve/pii/S1098301510751743. Accessed 20 May 2013.

59. Ertenli I (2012) PMS35 Cost-Effectiveness of Tocilizumab for the Treatment of Active Rheumatoid Arthritis (RA) Patients With Inadequate Response to Anti-TNF Treatment in Turkey. Value Heal 15: A445. Available: http://linkinghub.elsevier.com/retrieve/pii/S1098301512031038. Accessed 20 May 2013.

60. Farahani P, Goeree R, Gaebel K, Levine M (2005) Par16 Potential Problems in Using Rct Data To Estimate Cost-Effectiveness: Results From an Analysis of Etanercept Use in Rheumatoid Arthritis. Value Heal 8: A28–A29. Available: http://linkinghub.elsevier.com/retrieve/pii/S1098301510672257. Accessed 20 May 2013.

61. Fernandes R a., Boada V, Mould JF (2011) PMS32 Economic Analysis of Etanercept in Rheumatoid Arthritis from a Public perspective in Venezuela. Value Heal 14: A308. Available: http://linkinghub.elsevier.com/retrieve/pii/S1098301511019802. Accessed 20 May 2013.

62. Fernandes R a., Takemoto MLS, Tolentino a. CM, Takemoto MMS, Santos PML, et al. (2011) PMS40 Economic Evaluation of Etanercept in Rheumatoid Arthritis from the Public Payer perspective in Brazil. Value Heal 14: A309. Available: http://linkinghub.elsevier.com/retrieve/pii/S1098301511019887. Accessed 20 May 2013.

63. Fernandes R a., Tovar D a. B, Mould JF (2011) PMS36 Economic Analysis of Etanercept in Rheumatoid Arthritis from a Public perspective in Colombia. Value Heal 14: A308–A309. Available: http://linkinghub.elsevier.com/retrieve/pii/S109830151101984X. Accessed 20 May 2013.

64. Fernández I, Varela C, Badia X, Carreño Á, Polanco C (2008) Pms37 a Microsimulation-Based Cost-Utility Analysis of Rituximab (Mabthera ®) in Patients With Rheumatic Arthritis in Spain. Value Heal 11: A548. Available: http://linkinghub.elsevier.com/retrieve/pii/S1098301510668064. Accessed 20 May 2013.

65. Finckh A, Bansback N, Liang MH (2010) Cost-Effectiveness of Biologics in Early Rheumatoid Arthritis. Ann Intern Med 152: 334–335. Available: http://www.ncbi.nlm.nih.gov/pubmed/23679336.

66. Fleurence R, Spackman E (2006) Cost-effectiveness of biologic agents for treatment of autoimmune disorders: Structured review of the literature. J Rheumatol 33: 2124–2131. Available: http://www.scopus.com/inward/record.url?eid=2-s2.0-33751016675&partnerID=40&md5=71a852c403d00a51c1577166ad46cfaf.

67. Furneri G, Mantovani LG, Belisari A, Mosca M, Cristiani M, et al. (2012) Systematic literature review on economic implications and pharmacoeconomic issues of rheumatoid arthritis. Clin Exp Rheumatol 30: S72–S84. Available: http://www.scopus.com/inward/record.url?eid=2-s2.0-84870288967&partnerID=40&md5=aabb0cda5d5b4093364558ed7ba63fab.

68. Gabriel SE, Coyle D, Moreland LW (2001) A clinical and economic review of disease-modifying antirheumatic drugs. Pharmacoeconomics 19: 715–728. Available: http://www.scopus.com/inward/record.url?eid=2-s2.0-0034925015&partnerID=40&md5=cf7790cd3d1a0ed9ca7273a6a0422a60.

69. Gandhi P, Spooner J, Viraparia P, Kauf T (2008) Pms14 a Cost-Effectiveness Analysis of Biological Agents for Patients With Moderate To Severe Rheumatoid Arthritis Following Inadeqaute Response To Methotrexate. Value Heal 11: A258–A259. Available: http://linkinghub.elsevier.com/retrieve/pii/S1098301510708160. Accessed 20 May 2013.

70. Gao X, Hwang S, Carpiuc K, Stephens J, Sato R, et al. (2008) Pms70 Cost-Effectiveness of Etanercept Vs. Rituximab in the Treatment of Active Rheumatoid Arthritis in Colombia. Value Heal 11: A543. Available: http://linkinghub.elsevier.com/retrieve/pii/S1098301510667915. Accessed 20 May 2013.

71. Gao X, Hwang S, Carpiuc K, Stephens J, Sato R, et al. (2009) Pms22 Cost-Effectiveness of the Use of Etanercept Vs. Rituximab in Patients With Rheumatoid Arthritis in Mexico. Value Heal 12: A67. Available: http://linkinghub.elsevier.com/retrieve/pii/S1098301510733982. Accessed 20 May 2013.

72. Gossec L, Goupille P, Saraux a, Bregman B, Boccard E, et al. (2008) Pms21 Cost-Effectiveness Simulation Model of Abatacept Versus Rituximab in Rheumatoid Arthritis in France. Value Heal 11: A542–A543. Available: http://linkinghub.elsevier.com/retrieve/pii/S1098301510667897. Accessed 20 May 2013.

73. Guh D (2003) Cost-effectiveness of anakinra plus methotrexate versus methotrexate alone for the treatment of rheumatoid arthritis. Ann Rheum Dis 48: S264.

74. Guijarro P, Crespo C, Brosa M (2012) PMS49 Cost-Effectiveness Analysis of Etanercept and Infliximab in the Treatment of Rheumatoid Arthritis (RA) in Spain. Value Heal 15: A448. Available: http://linkinghub.elsevier.com/retrieve/pii/S1098301512031178. Accessed 20 May 2013.

75. Gulacsi L, Pentek M, Majer I (2004) Infliximab (REMICADE) in patients with rheumatoid arthritis; systematic review of the clinical and economic literature.

76. Hallinen T, Soini E, Eklund K, Puolakka K (2009) Pms58 Cost-Utility Analysis of Different Treatment Strategies After the Failure of First Biologic Treatment in Rheumatoid Arthritis (Ra) in the Finnish Setting. Value Heal 12: A444. Available: http://linkinghub.elsevier.com/retrieve/pii/S1098301510751962. Accessed 20 May 2013.

77. Haraoui B, Russell A, Thorne C (2007) Cost-Effectiveness Of Abatacept Versus Other Biologic Agents In Dmards And Anti-Tnf Inadequate Responders For The Management Of Moderate To Severe Rheumatoid Arthritis In Canada. J Rheumatol 34: 1628.

78. Hawkins N, Minda K, Parry D, Hines P, Yuan Y, et al. (2007) Par23 Cost-Utility of Abatacept, a New Biologic Treatment for Patients With Rheumatoid Arthritis Who Failed Anti-Tnf Therapy. Value Heal 10: A250. Available: http://linkinghub.elsevier.com/retrieve/pii/S1098301510649388. Accessed 20 May 2013.

79. Hernandez-Cruz B, Ariza-Ariza R, Rubio-Terres C, Navarro-Sarabia F (2004) Etanercept versus infliximab-methotrexate in the treatment of Spanish patients with rheumatoid arthritis refractory to disease-modifying anti-rheumatic drugs: A cost-effectiveness evaluation. Ann Rheum Dis 63: 510.

80. Kaczor MP, Wjcik R (2007) An economic analysis of TNF-alfa antagonists for rheumatoid arthritis in Polish settings.

81. Kamal KM, Miller L-A, Kavookjian J, Madhavan S (2006) Alternative Decision Analysis Modeling in the Economic Evaluation of Tumor Necrosis Factor Inhibitors for Rheumatoid Arthritis. Semin Arthritis Rheum 36: 50–60. Available: http://www.scopus.com/inward/record.url?eid=2-s2.0-33746467033&partnerID=40&md5=21bd89ccc8ff8da0af7959072cc36dcc.

82. Kamal K, Miller L, Madhavan S, Kavookjian J (2005) Par8 Alternative Decision Analysis Modeling in the Economic Evaluation of Tumor Necrosis Factor Inhibitors in Patients With Rheumatoid Arthritis. Value Heal 8: 342. Available: http://linkinghub.elsevier.com/retrieve/pii/S1098301510629026. Accessed 20 May 2013.

83. Kavanaugh A (2005) Cost effectiveness of TNF inhibitors in early rheumatoid arthritis (RA); analysis of infliximab plus methotrexate (MTX) compared with etanercept monotherapy. Ann Rheum Dis 50: S377.

84. Kavanaugh A, Patel KK, Bala M (2000) Cost effectiveness of infliximab in rheumatoid arthritis. Arthritis Rheum 43: S144.

85. Kielhorn a, Alvarez C, Aristides M, Diamantopoulos a (2006) Par8 Using Mabthera in Patients With Rheumatic Arthritis in Spain: Results of Cost-Effectiveness Data Based on Micro-Simulation. Value Heal 9: A26. Available: http://linkinghub.elsevier.com/retrieve/pii/S1098301510643896. Accessed 20 May 2013.

86. Kielhorn a, Rubbert a, Porter D, De Vita S, Brown B, et al. (2006) Par10 Cost-Effectiveness of Rituximab Therapy for Rheumatoid Arthritis: a Pan-European Analysis. Value Heal 9: A27. Available: http://linkinghub.elsevier.com/retrieve/pii/S1098301510643914. Accessed 20 May 2013.

87. Kievit W, Adang EM, Fransen J, Kuper HH, Van De Laar MAFJ, et al. (2008) The effectiveness and medication costs of three anti-tumour necrosis factor α agents in the treatment of rheumatoid arthritis from prospective clinical practice data. Ann Rheum Dis 67: 1229–1234. Available: http://www.scopus.com/inward/record.url?eid=2-s2.0-50249107555&partnerID=40&md5=a65865b99837e5c6fb065a58786d1aa8.

88. Kobelt G, Lindgren P, Geborek P (2009) Costs and outcomes for patients with rheumatoid arthritis treated with biological drugs in Sweden: A model based on registry data. Scand J Rheumatol 38: 409–418. Available: http://www.scopus.com/inward/record.url?eid=2-s2.0-70450164068&partnerID=40&md5=8f706ab8d4acd900e84bddf2896b0aeb.

89. Kobelt G, Lindgren P, Lindroth Y, Jacobson L, Eberhardt K (2005) Modelling the effect of function and disease activity on costs and quality of life in rheumatoid arthritis. Rheumatology (Oxford) 44: 1169–1175. Available: http://www.ncbi.nlm.nih.gov/pubmed/15956093. Accessed 18 March 2013.

90. Krieckaert CLM, Nair SC, Nurmohamed MT, van Dongen CJJ, Lems WF, et al. (2012) Evaluating the Cost-Effectiveness of Personalized Treatment with Adalimumab Using Serum Drug Levels in Rheumatoid Arthritis Patients. Arthritis Rheum 64: S779.

91. Kulp W, Corzillus M, Greiner W, Pientka L, Siebert U, et al. (2005) Influence of tumor necrosis factor alpha in rheumatoid arthritis. GMS Health Technol Assess 1: 1–8.

92. Kulp W, Greiner W, Graf von der Schulenburg JM (2003) [Cost effectiveness of pharmaceutical innovations. Health economic aspects of rheumatoid arthritis treatment with TNF-alpha antagonists]. Pharm Unserer Zeit 32: 420–425. Available: http://www.ncbi.nlm.nih.gov/pubmed/14526463. Accessed 20 May 2013.

93. Leardini G (2003) Cost-effectiveness analysis of etanercept versus infliximab and anakinra in the treatment of rheumatoid arthritis in Italy. Arthritis Rheum 48: S460–S461.

94. Lebmeier M, Pericleous L, Drost P, Dequen P, Ouwens M, et al. (2010) Pms37 the Cost-Effectiveness of Abatacept in Combination With Methotrexate for the Treatment of Patient With Active Rheumatoid Arthritis After an Inadequate Response To Methotrexate in the United Kingdom. Value Heal 13: A309. Available: http://linkinghub.elsevier.com/retrieve/pii/S1098301511721908. Accessed 20 May 2013.

95. Lee EK, Ko S, Bae S, Sung Y (2009) Cost-effectiveness of early use of etanercept in the treatment of rheumatoid arthritis.

96. Lekander I, Borgström F, Carli C, Svarvar P, Ljung T, et al. (2008) Ar2 a Cost Effectiveness Analysis of Infliximab Treatment in Patients With Rheumatoid Arthritis (Ra) in Sweden, Based on Data From the Sture Registry. Value Heal 11: A356. Available: http://linkinghub.elsevier.com/retrieve/pii/S1098301510662250. Accessed 21 May 2013.

97. Lewis G, Porter D, Brown B, Diamantopoulos A, Kielhorn A (2006) Economic Consequences Of Providing Rituximab As A New Therapeutic Option For Rheumatoid Arthritis In The Uk. Ann Rheum Dis 65: 283.

98. Lewis G, Kielhorn a, Diamantopoulos a (2007) Par22 Cost-Effectiveness of Rituximab (Mabthera) Compared With Abatacept (Orencia) for the Treatment of Moderate/Severe Rheumatoid Arthritis (Ra) in the United Kingdom. Value Heal 10: A249–A250. Available: http://linkinghub.elsevier.com/retrieve/pii/S1098301510649376. Accessed 21 May 2013.

99. Liao CH, Chen JJ, Pwu RF (2012) PMS16 Economic Evidence of Biologics in Rheumatoid Arthritis: A Systematic Review for Supporting Informed Decision of BNHI. Value Heal 15: A673. Available: http://linkinghub.elsevier.com/retrieve/pii/S1098301512021304. Accessed 21 May 2013.

100. Lopatriello S, Berto P (2003) Par4 Etanercept Versus Infliximab Plus Methotrexate in Rheumatoid Arthritis: a Cost-Effectiveness Analysis From the Italian Nhs Perspective. Value Heal 6: 725. Available: http://linkinghub.elsevier.com/retrieve/pii/S1098301510618499. Accessed 21 May 2013.

101. Lou S, Chen D, Cheng T, Huang C, Lin H, et al. (2008) Pms11 Cost-Effectiveness Analysis of Rituximab for Rheumatoid Arthritis in Taiwan. Value Heal 11: A257–A258. Available: http://linkinghub.elsevier.com/retrieve/pii/S1098301510708135. Accessed 21 May 2013.

102. Lutz M, Cuesta G, Morales G (2011) PMS8 Cost-Effectiveness analysis of Etanercept versus Available anti-Tnf and Il-6 Blockers for Treating Rheumatoid Arthritis in Guatemala. Value Heal 14: A562–A563. Available: http://linkinghub.elsevier.com/retrieve/pii/S1098301511032414. Accessed 21 May 2013.

103. Lyseng-Williamson KA, Foster RH (2004) Infliximab: A Pharmacoeconomic Review of its Use in Rheumatoid Arthritis. Pharmacoeconomics 22: 107–132. Available: http://www.scopus.com/inward/record.url?eid=2-s2.0-1242272199&partnerID=40&md5=09bd7c184f6893c75a389920ee3d28f0.

104. Lyseng-Williamson K a, Plosker GL (2004) Etanercept: a pharmacoeconomic review of its use in rheumatoid arthritis. Pharmacoeconomics 22: 1071–1095. Available: http://www.ncbi.nlm.nih.gov/pubmed/23072761.

105. Maetzel A (2008) Estimating the cost-effectiveness of lifelong infliximab for patients with rheumatoid arthritis (RA) in Canada. J Rheumatol 30: 1883.

106. Maier-Moldovan M, Yuan Y, Maclean R, L’Italien G (2008) Pms9 Cost-Effectiveness of Abatacept in Patients With Active Rheumatoid Arthritis (Ra)and Inadequate Response To Methotrexate (Mtx) Ortumor Necrosis Factor-Alpha Inhibitors (Anti-Tnfs):a Canadian Perspective. Value Heal 11: A257. Available: http://linkinghub.elsevier.com/retrieve/pii/S1098301510708111. Accessed 21 May 2013.

107. Majer I, Pentek M, Brodszky V, Gulacsi L (2006) Health economics modelling: costs, effectiveness and cost‐effectiveness of biological agents in rheumatoid arthritis with particular emphasis on infliximab (REMICADE) therapy: 1–168.

108. Malone DC, Ortmeier BG (2003) Cost Effectiveness Analysis Of Etanercept Versus Infliximab In The Treatment Of Rheumatoid Arthritis Patients. Ann Rheum Dis 62: 353.

109. Malone DC (2001) Cost-Effectiveness Analysis Of Etanercept Monotherapy Versus Infliximab Plus Methotrexate In The Treatment Of Rheumatoid Arthritis. Arthritis Rheum 44: S322. doi:10.1007/SpringerReference_12606.

110. Malone D, Ortmeier B (2002) Cost effectiveness analysis of etanercept (ENBREL) versus infliximab (Remicade) in the treatment of rheumatoid arthritis patients. Arthritis Rheum 46.

111. Maniadakis N, Boumpas D, Kourlaba G, Christou P, Anargiros I, et al. (2011) PMS45 Cost-Utility and Budget Impact Analysis of Certolizumab Pegol Plus Methotrexate for the Treatment of Moderate-to-Severe Active Rheumatoid Arthritis in Greece. Value Heal 14: A310. Available: http://linkinghub.elsevier.com/retrieve/pii/S1098301511019930. Accessed 21 May 2013.

112. Mantovani L, De Portu S, Intorcia M, Olivieri I (2008) Pms39 Cost-Utility of Abatacept in Rheumatoid Arthritis Patients With an Insufficient Response or Intolerance To Anti-Tnf Therapy in Italy: a Probabilistic Sensitivity Analysis. Value Heal 11: A548. Available: http://linkinghub.elsevier.com/retrieve/pii/S1098301510668088. Accessed 21 May 2013.

113. Marin B, Preux P-M, Druet-Cabanac M, Vergnenègre A (2006) Analysis of the cost-effectiveness of infliximab in the treatment of rheumatoid arthritis. Rev Rhum 73: 970–976. Available: http://linkinghub.elsevier.com/retrieve/pii/S1169833006002523. Accessed 21 May 2013.

114. Merkesdal S, Ruof J, Mittendorf T, Mau W, Zeidler H (2002) Health economics research in the area of chronic polyarthritis. 29: 21–29.

115. Merkesdal S, Ruof J, Mittendorf T, Zeidler H (2004) Cost-effectiveness of TNF-alpha-blocking agents in the treatment of rheumatoid arthritis. Expert Opin Pharmacother 5.

116. Merkesdal S, Ruof J (2002) [Current aspects of cost effectiveness of TNF-alpha blocking agents in patients with rheumatoid arthritis]. Z Rheumatol 61 Suppl 2: II/29–32. Available: http://www.ncbi.nlm.nih.gov/pubmed/12491120. Accessed 21 May 2013.

117. Miadi-Fargier H, Fautrel B, Maravic M, Daures J, Ollivier A, et al. (2008) Pms19 Cost-Effectiveness of Using Etanercept As First Line in Severe and Highly Active Rheumatoid Arthritis (Ra). Value Heal 11: A541–A542. Available: http://linkinghub.elsevier.com/retrieve/pii/S1098301510667873. Accessed 21 May 2013.

118. Mistry B, McCormick J, Diamantopoulos A, Kielhorn A (2006) Par6 Cost-Utility Analysis of Rituximab As a New Therapeutic Option for Rheumatoid Arthritis Patients in Canada. Value Heal 9: A222. Available: http://linkinghub.elsevier.com/retrieve/pii/S1098301510632652. Accessed 21 May 2013.

119. Monteiro I, Cafe A, Encarnação R, Diamantopoulos A, Dejonckheere F (2012) PMS42 Cost-Utility of Tocilizumab Monotherapy in Methotrexate Intolerant/Contra-Indicated, Moderate/Severe Rheumatoid Arthritis Patients in Portugal. Value Heal 15: A447. Available: http://linkinghub.elsevier.com/retrieve/pii/S1098301512031105. Accessed 21 May 2013.

120. Mucino E, Rivas R, Zapata L (2008) Pms15 Cost-Effectiveness of the Treatment for Early Rheumatoid Arthritis in Mexico: Infliximab Vs. Adalimumab. Value Heal 11: A259. Available: http://linkinghub.elsevier.com/retrieve/pii/S1098301510708172. Accessed 21 May 2013.

121. Nahar IK, Shojania K, Marra C a, Alamgir AH, Anis AH (2003) Infliximab treatment of rheumatoid arthritis and Crohn’s disease. Ann Pharmacother 37: 1256–1265. Available: http://www.ncbi.nlm.nih.gov/pubmed/12921510. Accessed 8 April 2013.

122. National Institution for Health and Clinical Excellence (2009) Rheumatoid arthritis: National clinical guideline for management and treatment in adults.

123. Nguyen C, Bounthavong M, Mendes M, Christopher M, Tran J, et al. (2012) Erratum. Pharmacoeconomics 30.

124. Nguyen C, Mendes M, Bounthavong M, Christopher M, Morreale A (2010) Pms28 Cost-Utility Analysis of Tumor Necrosis Factor-Alpha Inhibitors for the Treatment of Rheumatoid Arthritis Using a Markov Model. Value Heal 13: A127–A128. Available: http://linkinghub.elsevier.com/retrieve/pii/S1098301510726164. Accessed 21 May 2013.

125. Nurmohamed MT, Dijkmans BAC (2005) Efficacy, tolerability and cost effectiveness of disease-modifying antirheumatic drugs and biologic agents in rheumatoid arthritis. Drugs 65: 661–694. Available: http://www.scopus.com/inward/record.url?eid=2-s2.0-16444377894&partnerID=40&md5=7d636b80c41c57f565e80710a20ade1f.

126. Olyaeemanesh A, Doaee S, Nejati M, Mobinizadeh M, Aboee P, et al. (2012) Effectiveness and safety of Etanercept in treatment of arthritis. HealthMED 6: 2709–2716. Available: http://www.scopus.com/inward/record.url?eid=2-s2.0-84868118720&partnerID=40&md5=9b9e75d3d30aadb408f130b0c05507dd.

127. Patel AM, Lupash D, Chew D, Levesque MC, Moreland LW (2011) Cost-effectiveness of biologics compared with disease-modifying antirheumatic drugs in rheumatoid arthritis. Curr Rheumatol Rep 13: 381–382. Available: http://www.scopus.com/inward/record.url?eid=2-s2.0-80755139567&partnerID=40&md5=b3a6bef9a5530f79f81e19eb6d73a16d.

128. Pericleous L, Lebmeier M (2011) PMS31 Cost-Effectiveness of Abatacept for the Treatment of Rheumatoid Arthritis (RA) After the Failure of a First TNF inhibitor in the United Kingdom. Value Heal 14: A307–A308. Available: http://linkinghub.elsevier.com/retrieve/pii/S1098301511019796. Accessed 21 May 2013.

129. Pham B, McMartin K, Bornstein M, Machado M, Bombardier C, et al. (2008) Pms13 Cost Effectiveness of Biologics for Rheumatoid Arthritis: a Systematic Review. Value Heal 11: A258. Available: http://linkinghub.elsevier.com/retrieve/pii/S1098301510708159. Accessed 21 May 2013.

130. Pichon-Riviere A, Augustovski F, Garcia Marti S, Bardach A, Lopez A, et al. (2008) Etanercept, Infliximab and Adalimumab for the treatment of rheumatoid arthritis.: 7–9.

131. Piha T, Meirelles EDS, Kuriki W, Miranda P a. (2012) PMS39 Cost-Effectiveness Analysis of Certolizumab Pegol in Patients With Rheumathoid Arthritis from a Brazilian Private Perspective. Value Heal 15: A446. Available: http://linkinghub.elsevier.com/retrieve/pii/S1098301512031075. Accessed 21 May 2013.

132. Plisko R, Krzystek J, Rys P, Russel-Szymczyk M, Szkultecka-Debek M, et al. (2008) Pms25 Cost-Effectiveness of Rituximab (Mabthera) Compared With Tnf Inhibitors for the Treatment of Rheumatoid Arthritis (Ra) in Poland. Value Heal 11: A544. Available: http://linkinghub.elsevier.com/retrieve/pii/S1098301510667940. Accessed 21 May 2013.

133. Pompen M, Diamantopoulos a, Kievit W, Moers R, Kielhorn a (2007) Md2 Economic Consequences of Providing Rituximab As Atreatment Alternative for Rheumatoid Arthritis in the Netherlands. Value Heal 10: A235–A236. Available: http://linkinghub.elsevier.com/retrieve/pii/S1098301510648978. Accessed 21 May 2013.

134. Porter D, Lewis G, Brown B, Diamantopoulos a, Kielhorn a (2006) Par4 Cost-Effectiveness Analysis of Rituximab As a New Therapeutic Option for Rheumatoid Arthritis in the Uk. Value Heal 9: A25. Available: http://linkinghub.elsevier.com/retrieve/pii/S1098301510643859. Accessed 21 May 2013.

135. Puolakka K, Blåfield H, Kauppi M, Luosujärvi R, Peltomaa R, et al. (2012) Cost-effectiveness modelling of sequential biologic strategies for the treatment of moderate to severe rheumatoid arthritis in Finland. Open Rheumatol J 6: 38–43. Available: http://www.scopus.com/inward/record.url?eid=2-s2.0-84860681286&partnerID=40&md5=2b0589361c54bd3667e74e25f14cfb9b.

136. Purcaru O, Taylor P, Emery P, Palmer S (2010) Pms42 Cost-Effectiveness of Certolizumab Pegol Plus Methotrexate or As Monotherapy for the Treatment of Active Rheumatoid Arthritis in the United Kingdom. Value Heal 13: A310. Available: http://linkinghub.elsevier.com/retrieve/pii/S1098301511721957. Accessed 21 May 2013.

137. Quintana G, Restrepo JP, Caceres H a., Rueda JD, Rosselli D (2011) Pms32 Economic Evaluation of the Treatment of Rheumatoid Arthritis With Anti-Tnf Biological Therapies in Colombia. Value Heal 14: A129. Available: http://linkinghub.elsevier.com/retrieve/pii/S1098301511008564. Accessed 21 May 2013.

138. Ravasio R, Lucioni C (n.d.) Economic evaluation of etanercept in AR [Valutazione economica di etanercept nel trattamento dell’Artrite Reumatoide]. 2006.

139. Romero M, Latorre M, Alvarado C, Karpf E, Alvis N (2011) QA2 Analisis De Costo-Utilidad De Rituximab Posterior Al Fallo Por Anti-Tnf En Artritis Reumatoide Para Colombia. Value Heal 14: A536. Available: http://linkinghub.elsevier.com/retrieve/pii/S1098301511030865. Accessed 21 May 2013.

140. Rubbert a, Kielhorn a, Schach S, Brown B (2006) Par5 Cost-Effectiveness of Rituximab As a New Treatment Modality for Rheumatoid Arthritis in Germany. Value Heal 9: A25. Available: http://linkinghub.elsevier.com/retrieve/pii/S1098301510643860. Accessed 21 May 2013.

141. Rubio-Terras C, Domanguez-Gil A (2001) Pharmacoeconomic analysis of the treatment of rheumatoid arthritis with Leflunomide in comparison with the combination of Infliximab and Methotrexate. J Med Econ 4.

142. Saggia M, Pina F (2006) Par1 Cost-Effectiveness Analysis of Etanercept Vs Infliximab for Treatment of Severe Rheumatoid Arthritis in Brazil. Value Heal 9: A220. Available: http://linkinghub.elsevier.com/retrieve/pii/S1098301510632603. Accessed 21 May 2013.

143. Sany J, Cohen JD, Combescure C, Bozonnat MC, Roch-Bras F, et al. (2009) Medico-economic evaluation of infliximab in rheumatoid arthritis--prospective French study of a cohort of 635 patients monitored for two years. Rheumatology (Oxford) 48: 1236–1241. Available: http://www.scopus.com/inward/record.url?eid=2-s2.0-70350769210&partnerID=40&md5=b606074275c30831a8aad63eda310aa6.

144. Saraux A (2008) Cost-effectiveness simulation model of abatacept versus anti-TNF treatment strategies in rheumatoid arthritis in France. Arthritis Rheum 58: S463.

145. Schulze-Koops H, Deeg M, Runge C, Volmer T, Brecht JG (2009) [Health-economic assessment of combination therapy for rheumatoid arthritis with methotrexat and etanercept based on the TEMPO Study]. Z Rheumatol 68: 836–841. Available: http://www.ncbi.nlm.nih.gov/pubmed/19756664. Accessed 21 May 2013.

146. Scott DL, Steer S (2009) NICE guidelines on anti-tumor necrosis factor therapy for RA. Nat Clin Pract Rheumatol 5: 16–17. Available: http://www.scopus.com/inward/record.url?eid=2-s2.0-58049215333&partnerID=40&md5=f307f0f7ab6d986baa6a81cb48a417af.

147. Scott DL, Kingsley G (2010) Clinical effectiveness of biologics in clinical practice. Arthritis Res Ther 12: 115. Available: http://www.pubmedcentral.nih.gov/articlerender.fcgi?artid=2888208&tool=pmcentrez&rendertype=abstract. Accessed 21 May 2013.

148. Smolen JS, Landewé R, Breedveld FC, Dougados M, Emery P, et al. (2010) EULAR recommendations for the management of rheumatoid arthritis with synthetic and biological disease-modifying antirheumatic drugs. Ann Rheum Dis 69: 964–975. Available: http://www.scopus.com/inward/record.url?eid=2-s2.0-77953696525&partnerID=40&md5=29080e6ab79712c35cd677a75b355623.

149. Soini E, Hallinen T, Kauppi M, Vihervaara V, Puolakka K (2010) Pms43 Cost-Utility and Multinomial Expected Value of Perfect Information of Sequenced Treatment With Biologics in Moderate-To-Severe Rheumatoid Arthritis: Analysis Based on Clinically Meaningful Responses. Value Heal 13: A310. Available: http://linkinghub.elsevier.com/retrieve/pii/S1098301511721969. Accessed 21 May 2013.

150. Soini E, Hallinen T, Taiha M, Honkanen V (2010) Bl1 Cost-Effectiveness, Value of Information, and Budget Impact of Certolizumab Pegol Compared To Subcutaneous Tumor Necrosis Factor (Tnf) Inhibitors and Methotrexate in the Treatment of Moderate-To-Severe Rheumatoid Arthritis in Finland. Value Heal 13: A243. Available: http://linkinghub.elsevier.com/retrieve/pii/S1098301511718629. Accessed 21 May 2013.

151. Solé J, Ortega G, Kielhorn a (2007) Par3 Cost-Benefit of Rituximab for Rheumatoid Arthritis Patients in Argentina. Value Heal 10: A243–A244. Available: http://linkinghub.elsevier.com/retrieve/pii/S1098301510649182. Accessed 21 May 2013.

152. Spalding J, Hay J (2004) Par4 Cost-Effectiveness of Tumor Necrosis Factor Alpha (Tnf-Alpha) Inhibitors As First-Line Agents in Rheumatoid Arthritis. Value Heal 7: 241. Available: http://linkinghub.elsevier.com/retrieve/pii/S1098301510621286. Accessed 21 May 2013.

153. Suarez- Almazor M, Ortiz Z, M L-O, Moffett M, Pak C, et al. (2007) Long-term Clinical and Cost-Effectiveness of Infliximab and Etanercept for Rheumatoid Arthritis.

154. Suarez-Almazor ME, Ortiz Z, Lopez-Olivo M de los A, Moffett M, Pak C, et al. (2007) Infliximab and Etanercept in Rheumatoid Arthritis: Systematic Review of Long-term Clinical Effectiveness, Safety, and Cost-effectiveness.

155. Suka M, Yoshida K (n.d.) Economic evaluation of a new treatment for rheumatoid arthritis. 2007.

156. Sullivan SD, Alfonso-Cristancho R, Carlson J, Mallya U, Ringold S (2013) Economic consequences of sequencing biologics in rheumatoid arthritis: a systematic review.

157. Symmons D, Tricker K, Roberts C, Davies L, Dawes P, et al. (2005) The British Rheumatoid Outcome Study Group (BROSG) randomised controlled trial to compare the effectiveness and cost-effectiveness of aggressive versus symptomatic therapy in established rheumatoid arthritis. 9.

158. Takemoto M, Fernandes R a., Fujii RK, Mould J, Tang B (2012) PSY25 Etanercept in Early Rheumatoid Arthritis: Economic Evaluation From the Public Payer Perspective in Brazil. Value Heal 15: A512–A513. Available: http://linkinghub.elsevier.com/retrieve/pii/S1098301512034614. Accessed 21 May 2013.

159. Tanaka E, Hoshi D, Nakajima A (2011) Pharmacoeconomic analysis of a humanized anti-interleukin-6 (IL-6) receptor monoclonal antibody, tocilizumab, in rheumatoid arthritis.

160. Tanaka E, Inoue E, Hoshi D, Kobayashi A, Naoki, et al. (2012) Evaluation of the Cost-Effectiveness of Rheumatoid Arthritis Treatment with Biologic Agents Using the IORRA Cohort Database. Arthritis Rheum 64: S779.

161. Taylor M, Trueman P, Reynolds A, P C (2007) Economic Benefits Of Etanercept Following Anti-Tnf Treatment In Patients With Rheumatoid Arthritis: An Analysis Using Tempo Data. Rheumatology (Oxford) 46: I105.

162. Taylor M, Righetti C, Conway P, Lebmeier M (2009) Economic Evaluation Of Therapeutic Approaches For Rheumatoid Arthritis: The Case For Sequential Anti-Tnf Use. Rheumatology 48: I105.

163. Taylor M, Conway P, Lebmeier M, Ganderton M (2008) Pms24 the Cost-Effectiveness of Sequential Use of Anti-Tumor Necrosis Factor Agents in the Treatment of Rheumatoid Arthritis. Value Heal 11: A544. Available: http://linkinghub.elsevier.com/retrieve/pii/S1098301510667939. Accessed 21 May 2013.

164. Tella MN, Feinglass J, Chang RW (2003) in rheumatology : a review of the literature , 2001 – 2002: 2001–2002.

165. Tosh JC, Wailoo AJ, Scott DL, Deighton CM (2011) Cost-effectiveness of combination nonbiologic disease-modifying antirheumatic drug strategies in patients with early rheumatoid arthritis. J Rheumatol 38: 1593–1600. Available: http://www.ncbi.nlm.nih.gov/pubmed/21572149. Accessed 21 May 2013.

166. Triggiani M (n.d.) Cost-effectiveness analysis of two biological treatments (Adalimumab vs. Etanercept) in moderate-severe rheumatoid arthritis [Analisi costo-efficacia di due strategie terapeutiche con farmaci biologici (Adalimumab vs. Etanercept) nell’artrite reumatoide d. 2006.

167. Tsao NW, Bansback NJ, Shojania K, Marra C a (2012) The issue of comparators in economic evaluations of biologic response modifiers in rheumatoid arthritis. Best Pract Res Clin Rheumatol 26: 659–676. Available: http://www.ncbi.nlm.nih.gov/pubmed/23218430. Accessed 21 May 2013.

168. Tsutani K, Igarashi A (2005) Anti-rheumatoid biologics and pharmacoeconomic evaluation.

169. Tsutani K, Igarashi A, Shiroiwa T (2010) Pharmacoeconomics of molecular target drugs in Japan 2010.

170. Valle-Mercado C, Cubides MF, Parra-Torrado M, Rosselli D (2011) PMS50 Cost-Effectiveness of Biologic Agents Compared with Methotrexate in the Treatment of Rheumatoid Arthritis in Colombia. Value Heal 14: A311. Available: http://linkinghub.elsevier.com/retrieve/pii/S109830151101998X. Accessed 21 May 2013.

171. Walsh CAE, Minnock P, Slattery C, Kennedy N, Pang F, et al. (2007) Quality of life and economic impact of switching from established infliximab therapy to adalimumab in patients with rheumatoid arthritis. Rheumatology 46: 1148–1152. Available: http://www.scopus.com/inward/record.url?eid=2-s2.0-34447307138&partnerID=40&md5=bf28beee7daedab461b43cb2efe8973c.

172. Van Der Velde G, Pham B, MacHado M, Ieraci L, Witteman W, et al. (2011) Cost-effectiveness of biologic response modifiers compared to disease-modifying antirheumatic drugs for rheumatoid arthritis: A systematic review. Arthritis Care Res 63: 65–78. Available: http://www.scopus.com/inward/record.url?eid=2-s2.0-79952219360&partnerID=40&md5=3ececd1877ce478462d3bd9fc47d152c.

173. Van Nooten F, Gajria K, Kansal a. R (2011) PMS38 A Structured Literature Review of Rheumatoid Arthritis Economic Models for Biologics. Value Heal 14: A309. Available: http://linkinghub.elsevier.com/retrieve/pii/S1098301511019863. Accessed 21 May 2013.

174. Vanoverbeke N, Annemans L, Cauchie P, Hendrickx E (2004) Par8 Health Economic Evaluation of Patients With Rheumatoid Arthritis (Ra) Treated With Adalimumab. Value Heal 7: 663. Available: http://linkinghub.elsevier.com/retrieve/pii/S1098301510657142. Accessed 21 May 2013.

175. Vargas-Valencia J, Sotelo-Guzmán M, Mould-Quevedo JF, Muciño-Ortega E, Galindo-Suarez RM (2011) Pms22 Cost-Effectiveness Analysis of Etanercept in the Treatment of Rheumatoid Arthritis in Mexico. Value Heal 14: A127. Available: http://linkinghub.elsevier.com/retrieve/pii/S1098301511008461. Accessed 21 May 2013.

176. Venson R, Wiens A, Correr CJ, Otuki MF (2011) Economic evaluation of anticitokines adalimumab, etanercept and infliximab for treatment of rheumatoid arthritis in ParanÃ¡ state, Brazil. Physis 21: 359–376.

177. Vera-Llonch M, Massarotti E, Shadick N, Wolfe F, Westhovens R, et al. (2007) Cost-Effectiveness Of Abatacept In Patients With Moderately To Severely Active Rheumatoid Arthritis And Inadequate Response To Methotrexate. Ann Rheum Dis 66: 277.

178. Villoro R, Hidalgo a., Ferro B, Talavera P (2011) PMS46 Cost-Utility Analysis of Certolizumab Pegol versus Alternative Tumor Necrosis Factor-Inhibitors, for the Treatment of Moderate-to-Severe Rheumatoid Arthritis in Spain. Value Heal 14: A310–A311. Available: http://linkinghub.elsevier.com/retrieve/pii/S1098301511019942. Accessed 21 May 2013.

179. Wolfe F, Michaud K (2010) The loss of health status in rheumatoid arthritis and the effect of biologic therapy: A longitudinal observational study. Arthritis Res Ther 12. Available: http://www.scopus.com/inward/record.url?eid=2-s2.0-77956985320&partnerID=40&md5=3213791ae2e457eed1640d3d19f84173.

180. Wong JB, Branco JC, Cobrado N, Nero P (2003) Cost-Effectiveness Of Lifelong Infliximab For Rheumatoid Arthritis In Portugal. Ann Rheum Dis 62: 360.

181. Wong J, Ballina J, Fernandez-Sueiro J, Al. E (2004) Cost-effectiveness of infliximab for rheumatoid arthritis in Spain. Ann Rheum Dis 63.

182. Wong JB, Breedveld FC, Smolen JS, Kavanaugh AF, Riel PL van, et al. (2001) Cost-effectiveness of 102-weeks of infliximab for rheumatoid arthritis. Arthritis Rheum 44: S311.

183. Wong JB, Singh G, Kavanaugh A (2000) Estimating the cost-effectiveness of 54 weeks of infliximab for rheumatoid arthritis. Arthritis Rheum 43: S144.

184. Vorobyev P, Lesnicheva M, Avksentyeva M, Alekseyeva Y, Karateyev D (2010) Pms23 Economical Evaluation of Adalimumab in Patients With Rheumatoid Arthritis. Value Heal 13: A126–A127. Available: http://linkinghub.elsevier.com/retrieve/pii/S1098301510726115. Accessed 21 May 2013.

185. Yen J-H (2006) Treatment of early rheumatoid arthritis in developing countries. Biologics or disease-modifying anti-rheumatic drugs? Biomed Pharmacother 60: 688–692. Available: http://www.scopus.com/inward/record.url?eid=2-s2.0-33751534165&partnerID=40&md5=3abd5c4f1c63cac1598894adcdc15981.

186. Yuan Y, Maier-Moldovan M, Maclean R, L’Italien G (2008) Pms10 the Cost-Effectiveness of Abatacept Versus Rituximab in Patients With Rheumatoid Arthritis: a Perspective of the Canadian Publicly Funded Health Care System. Value Heal 11: A257. Available: http://linkinghub.elsevier.com/retrieve/pii/S1098301510708123. Accessed 21 May 2013.

187. Yunger S, Mistry B (2009) Pms34 an Exploratory Evaluation of the Cost-Effectiveness of Rituximab and Abatacept in the Treatment of Moderate To Severe Rheumatoid Arthritis After an Inadequate Response To a Tumour Necrosis Factor Inhibitor in Canada. Value Heal 12: A70. Available: http://linkinghub.elsevier.com/retrieve/pii/S1098301510734100. Accessed 21 May 2013.

188. Zhang Y, Yang L, Dong P, Xie X, Tang B (2012) PMS14 Cost-Effectiveness Analysis of Etanercept in the Treatment of Rheumatoid Arthritis in China. Value Heal 15: A673. Available: http://linkinghub.elsevier.com/retrieve/pii/S1098301512021286. Accessed 21 May 2013.

189. Zou D, Desjardins O, Tsao N, Goeree R (2010) Pms29 Canadian Cost-Effectiveness Analysis of Abatacept (Orencia^TM^) for the Management of Moderately To Severely Active Rheumatoid Arthritis in Patients With Inadequate Response To Methotrexate. Value Heal 13: A128. Available: http://linkinghub.elsevier.com/retrieve/pii/S1098301510726176. Accessed 21 May 2013.
