# Supplementary material for: The Cost-Effectiveness of Biologics for the Treatment of Rheumatoid Arthritis: A Systematic Review
Source: PLoS One. 2015 Mar 17;10(3):e0119683. doi: 10.1371/journal.pone.0119683 (PMC4363598; doi:10.1371/journal.pone.0119683)
Supplement: S1 Table — (DOCX) [file pone.0119683.s004.docx]

**S1 Table. Inclusion and exclusion criteria.**

|  | Inclusion | Exclusion |
| --- | --- | --- |
| Patients (P) | Rheumatoid arthritis |  |
| Intervention (I) | Biologics (ABT, ADA, ANA, CER, ETN, GOL, IFX, RTX, TOC) as a monotherapy or combined with cDMARDs |  |
| Control (C) | cDMARD(s) or other biologic(s) | No treatment or placebo |
| Outcome (O) | QALYs  At least direct healthcare costs  ICER (if applicable) | No QALYs reported  No costs reported |
| Study (S) | CUA  Modeling, empiric or observational analysis | CEA without QALY as the effectiveness measures  Other study designs  Published only as an abstract  No English full-text |
| ABT=abatacept, ADA=adalimumab, ANA=anakinra, cDMARD=conventional disease-modifying antirheumatic drugs, CEA=cost-effectiveness analysis, CER=certolizumab pegol, CUA=cost-utility analysis, ETN=etanercept, GOL=golimumab, ICER=incremental cost-effectiveness ratio, IFX=infliximab, QALY=quality-adjusted life year, RTX=rituximab, TOC=tocilizumab | | |
